# Association of a cytarabine chemosensitivity related gene expression signature with survival in cytogenetically normal acute myeloid leukemia

**DOI:** 10.18632/oncotarget.13650

**Published:** 2016-11-26

**Authors:** Han Yan, Lu Wen, Dan Tan, Pan Xie, Feng-mei Pang, Hong-hao Zhou, Wei Zhang, Zhao-qian Liu, Jie Tang, Xi Li, Xiao-ping Chen

**Affiliations:** ^1^ Department of Clinical Pharmacology, Xiangya Hospital, Central South University, Changsha 410008, P. R. China; ^2^ Institute of Clinical Pharmacology, Central South University, Hunan Key Laboratory of Pharmacogenetics, Changsha 410078, P. R. China; ^3^ Department of Diagnostic Radiology, Hunan Cancer Hospital, Changsha 410013, Hunan, P. R. China; ^4^ Hunan Province Cooperation Innovation Center for Molecular Target New Drug Study, Hengyang 421001, P. R. China

**Keywords:** cytarabine, chemosensitivity, acute myeloid leukemia, prognosis, signature

## Abstract

The prognosis of cytogenetically normal acute myeloid leukemia (CN-AML) varies greatly among patients. Achievement of complete remission (CR) after chemotherapy is indispensable for a better prognosis. To develop a gene signature predicting overall survival (OS) in CN-AML, we performed data mining procedure based on whole genome expression data of both blood cancer cell lines and AML patients from open access database. A gene expression signature including 42 probes was derived. These probes were significantly associated with both cytarabine half maximal inhibitory concentration values in blood cancer cell lines and OS in CN-AML patients. By using cox regression analysis and linear regression analysis, a chemo-sensitive score calculated algorithm based on mRNA expression levels of the 42 probes was established. The scores were associated with OS in both the training sample (*p*=5.13 × 10^−4^, HR=2.040, 95% CI: 1.364-3.051) and the validation sample (*p*=0.002, HR=2.528, 95% CI: 1.393-4.591) of the GSE12417 dataset from Gene Expression Omnibus. In The Cancer Genome Atlas (TCGA) CN-AML patients, higher scores were found to be associated with both worse OS (*p*=0.013, HR=2.442, 95% CI: 1.205-4.950) and DFS (*p*=0.015, HR=2.376, 95% CI: 1.181-4.779). Results of gene ontology (GO) analysis showed that all the significant GO Terms were correlated with cellular component of mitochondrion. In summary, a novel gene set that could predict prognosis of CN-AML was identified presently, which provided a new way to identify genes impacting AML chemo-sensitivity and prognosis.

## INTRODUCTION

Acute myeloid leukemia (AML), characterized by the rapid growth of abnormal white blood cells interfering with the production of normal blood cells, is the most common type of acute leukemia affecting adults. Presently, induction chemotherapy with cytarabine and anthracyclines is the first-line treatment for AML except for acute promyelocytic leukemia (According to NCCN AML Guidelines 2015, version 1). However, outcomes of AML vary greatly among patients after chemotherapy. Clinical studies have shown that the five-year survival rate of AML varies from 18% to 82%, and relapse rate varies from 33% to 80% [1–4].

It is well known that chromosomal abnormalities are major prognostic factors in AML. Based on karyotype, AML patients can be divided into favorable, intermediate and unfavorable cytogenetics risk groups which show different survival profiles [5]. However, the detailed mechanism of the prognosis variation in normal karyotype AML (CN-AML) patients, the most common type, remains unclear. Several factors affecting AML prognosis have been identified, including somatic mutations in genes such as *NPM1, FLT3-ITD, CEBPA, WT1, ASXL1, IDH1/2, DNMT3A*, and *RUNX1* [6–10]. Moreover, expression levels of *LFE1* [11], *CXXC5* [12], *EVL1, MEL1* [13], and miR-9* [14] are also reported to be associated with AML prognosis. However, only a small part of AML prognosis variation can be explained by these above-mentioned factors.

Cancer cell lines, which were descended from naturally occurring tumors, are commonly used for molecular biology research and drug discovery *in vitro*. Previous studies have confirmed that the use of pharmacological data and genomic information of cancer cell lines can help researchers to identify therapeutic biomarkers [15–17]. These data are also helpful to find genomic variants associated with sensitivity of anti-tumor drugs and thus potentially affect cancer prognosis. Cytarabine is a first-line drug for inducing remission in common therapeutic schedule for AML. The achievement of complete remission (CR) is indispensable for a better prognosis; meanwhile, chemo-resistance is essential for treatment failure and poor outcomes for AML [18–20]. Therefore, further identification of genomic information affecting drug response phenotype in leukemia cell lines may help to find new factors affecting AML prognosis.

Recently, a lot of classified studies based on RNA microarray have been performed to establish the classifier which might help to predict the outcomes of CN-AML. For example, Metzeler *et al.* reported an 86-probe-set mRNA expression signature which was correlated with overall survival (OS) of AML [21]. Gentles *et al.* found a leukemic stem cell gene expression signature correlated with clinical outcomes in AML [22]. Garzon *et al.* derived a lncRNA score composed of 48 lncRNAs and found that the score was an independent marker for the outcome in CN-AML patients [23]. However, no studies have focused on genes related to chemo-sensitivity. In 2013, an open access database named Genomics of Drug Sensitivity in Cancer (GDSC) was developed [24]. This database includes date of half maximal inhibitory concentration (IC_50_) values for 138 anticancer drugs on more than 800 cancer cell lines. Whole genome mutations and expression levels are also available for the GDSC database. To find out whether genes correlated with chemo-sensitivity could also predict the outcome of AML, we downloaded IC_50_ values of cytarabine from 96 leukemia cell lines and raw whole genome expression data of these cell lines from GDSC in this study. Probes significantly associated with IC_50_ levels in the cells and OS in GSE12417 dataset, a whole genome expression dataset for CN-AML in NCBI Gene Expression Omnibus (GEO), were selected. Based on the selected probes, chemo-sensitivity score was derived and replicated in a TCGA CN-AML dataset.

## RESULTS

### Cluster analysis in blood cell lines

**Figure 1:**
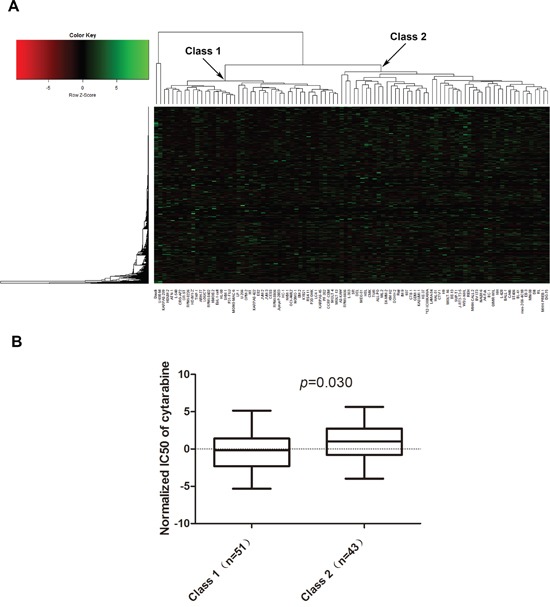
Whole genome expression pattern impact cytarabine sensitivity in blood cancer cell lines **A.** Cluster analysis of all probes in blood cancer cell lines, **B.** Comparison of cytarabine IC_50_ values between two classes of blood cancer cell lines divided by cluster analysis.

At first, we conducted a cluster analysis based on expression of all probes. The blood cancer cell lines were clustered into two classes except for Daudi (a human Burkitt's lymphoma cell line) and U-698-M (a B-Acute Lymphoblastic Leukemia cell line) (Figure 1A). Significant difference in mean cytarabine IC_50_ values between the two classes was observed (*p*=0.030, Figure 1B). These results indicated that the whole genome expression pattern can affect cytarabine sensitivity in blood cell lines.

### Function prediction of genes notably impacting cytarabine IC_50_ in blood cancer cell lines

**Figure 2:**
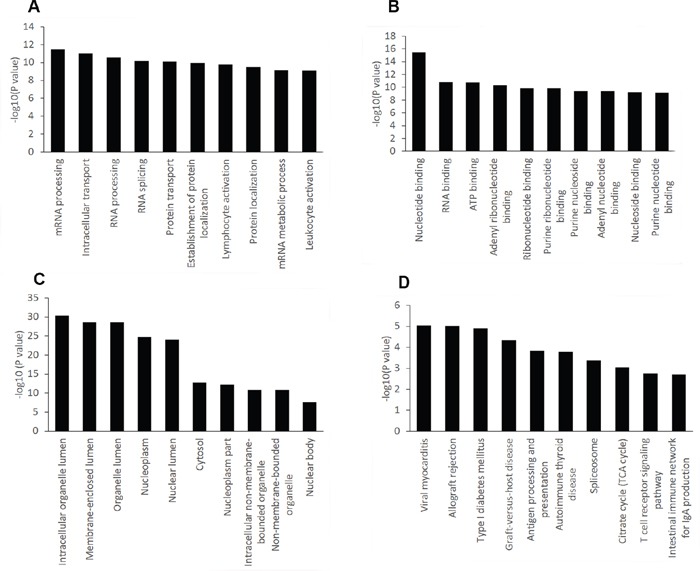
GO and KEGG pathway analysis of genes notably impacting cytarabine IC50 in blood cancer cell lines The top ten significantly enriched GO categories and pathways were calculated and plotted as the − 1 × log10 (*p* value). **A.** Biological process, **B.** Molecular function, **C.** Cellular component, **D.** KEGG Pathways.

Results of linear-regression analysis showed that 4207 probes were significantly associated with cytarabine IC_50_ values (raw *p*<0.05) in the GDSC blood cell lines. GO annotation was carried out to predict the potential function of genes targeted by the probes. In terms of biological processes, 4 of the top 10 categories belonged to RNA processing modification and metabolic process (Figure 2A). Interestingly, molecular functions of all top 10 categories were related to protein or nucleic acid binding (Figure 2B). With regard to cellular components, lumen appeared in 3 most enriched categories (Figure 2C). KEGG database was also used to identify gene networks affected by these genes (Figure 2D). Most pathways were related to immune system. The details of pathway analysis based on all 4207 probes were listed in supplementary materials (Supplementary material S2).

### A 42 probe set correlated with both cytarabine sensitivity and AML OS

Among the 4207 probes associated with cytarabine IC_50_ values, 453 were significantly associated with OS in GSE12417 U133 AB sample, while 337 were associated with OS in GSE12417 U133 plus sample (univariate cox regression analysis *P*<0.05). Finally, 42 probes correlated with both cytarabine sensitivity and OS in the same direction were identified. Details of these probes were listed in Table 1. After analysis in David database, 33 GO Terms with raw *p*<0.05 were identified, and four (GO:0044429, Bonferroni *p*=1.74 × 10^−5^; GO:0005759, Bonferroni *p*=0.002; GO:0031980, Bonferroni *p*=0.002; GO:0005739, Bonferroni *p*=0.006) of them passed correction for multiple tests (Table 2). Interestingly, all these four GO Terms correlated with cellular component of mitochondrion which plays important roles in AML progression.

**Table 1:** Statistical analysis results for the 42 probes in blood cancer cell lines and in the GSE12417 dataset

Gene	Probe name	GDSC Blood cancer cell lines		GSE12417				Prognostic Index
		p value	t value	U133AB sample		U133 Plus sample		
				p value	Z value	p value	Z value	
ULK1	209333_at	1.44E-05	−4.578	0.045	−2.004	0.018	−2.362	−0.309
PIGF	205078_at	2.54E-05	4.431	0.043	2.020	0.041	2.045	0.193
ABCC5	209380_s_at	9.26E-05	−4.085	0.011	−2.541	0.035	−2.109	−0.336
MRPL40	203152_at	9.84E-05	4.069	0.003	2.959	0.018	2.366	0.253
DARS2	218365_s_at	1.60E-04	3.934	0.046	1.992	0.011	2.532	0.193
ITSN1	35776_at	2.27E-04	−3.835	0.027	−2.208	0.041	−2.041	−0.274
ZC3HAV1	213051_at	4.09E-04	−3.665	0.032	−2.149	0.017	−2.397	−0.355
NDUFB5	203621_at	5.32E-04	3.588	0.003	3.022	0.004	2.865	0.341
SKAP2	204361_s_at	7.54E-04	3.483	0.025	2.243	0.038	2.072	0.186
ZNF451	215012_at	0.001	−3.367	0.038	−2.070	0.005	−2.821	−0.316
IARS2	217900_at	0.002	3.197	0.010	2.562	0.018	2.358	0.212
MPP1	202974_at	0.003	−3.049	0.018	−2.367	0.044	−2.010	−0.363
ZNF259P1/ZPR1	217185_s_at	0.004	2.961	0.003	3.023	0.011	2.541	0.231
BCL2L1	212312_at	0.004	−2.916	0.022	−2.288	0.042	−2.032	−0.309
CHMP4A/TM9SF1	218572_at	0.006	2.836	0.026	2.219	0.047	1.987	0.197
GAS2	205848_at	0.007	2.744	0.029	2.185	0.001	3.403	0.191
ME2	210154_at	0.008	2.715	0.021	2.307	0.016	2.412	0.221
ALCAM	201951_at	0.008	2.710	0.000	3.634	0.000	5.064	0.363
DNAJC1	218409_s_at	0.009	2.675	0.005	2.791	0.023	2.282	0.259
VDAC1	212038_s_at	0.009	2.673	0.005	2.804	0.018	2.376	0.213
SLC25A38	217961_at	0.009	−2.648	0.011	−2.540	0.016	−2.420	−0.314
IL6R	205945_at	0.010	2.636	0.003	2.986	0.022	2.282	0.260
ME2	210153_s_at	0.012	2.568	0.018	2.367	0.002	3.053	0.254
ETFDH	33494_at	0.013	2.527	0.040	2.058	0.043	2.020	0.158
TAL1	206283_s_at	0.017	−2.441	0.036	−2.092	0.027	−2.216	−0.339
TAL1	216925_s_at	0.017	−2.427	0.011	−2.541	0.032	−2.147	−0.381
BMP2K	219546_at	0.017	−2.420	0.013	−2.486	0.033	−2.137	−0.308
FH	203033_x_at	0.019	2.379	0.016	2.399	0.033	2.131	0.213
SLC14A1	205856_at	0.021	−2.353	0.037	−2.091	0.048	−1.974	−0.356
SPATS2	218324_s_at	0.021	2.338	0.023	2.268	0.017	2.391	0.272
P4HTM	222125_s_at	0.026	2.269	0.013	2.478	0.029	2.184	0.212
PLA2G4A	210145_at	0.031	2.195	0.007	2.689	0.023	2.281	0.242
TRIB2	202478_at	0.031	−2.184	0.030	−2.174	0.033	−2.126	−0.310
ACYP2/LOC101927144	206833_s_at	0.032	2.182	0.008	2.636	0.030	2.167	0.321
SYNCRIP	217834_s_at	0.033	2.166	0.025	2.239	0.007	2.691	0.211
IDH3A	202069_s_at	0.033	2.161	0.012	2.509	0.014	2.466	0.215
TPD52	201688_s_at	0.033	2.161	0.004	2.895	0.008	2.662	0.268
HIST1H2APS4	216585_at	0.033	2.160	0.038	2.072	0.005	2.795	0.229
ERMP1	218342_s_at	0.036	2.124	0.034	2.119	0.038	2.072	0.217
SLC25A37	221920_s_at	0.038	−2.099	0.016	−2.413	0.027	−2.213	−0.368
TCTN3	212121_at	0.046	2.026	0.000	3.506	0.009	2.620	0.331
CD164	208654_s_at	0.048	2.008	0.012	2.519	0.006	2.732	0.293

**Table 2:** Four most significantly GO terms for the selected 42 probes

Category	GO Term	Count	%	*p* value	Bonferroni
GOTERM_CC_FAT	GO:0044429 ~ mitochondrial part	12	30.0	1.54E-07	1.74E-05
GOTERM_CC_FAT	GO:0005759 ~ mitochondrial matrix	7	17.5	2.18E-05	0.002
GOTERM_CC_FAT	GO:0031980 ~ mitochondrial lumen	7	17.5	2.18E-05	0.002
GOTERM_CC_FAT	GO:0005739 ~ mitochondrion	12	30.0	5.36E-05	0.006

### Association of the chemo-sensitivity score based on 42 probes with AML survival in patients from GSE12417 dataset

**Figure 3:**
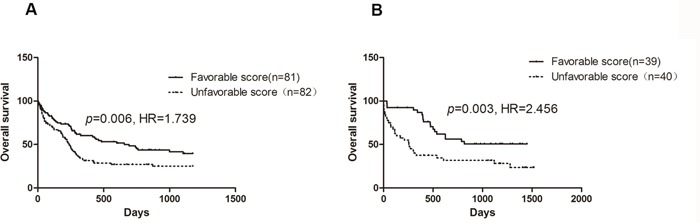
Survival curve of AML patients stratified by chemo-sensitivity score **A.** U133 AB samples; **B.** U133 plus samples.

The detailed information including univariate cox score and combined cox score of the 42 selected probes mapped to 44 annotated genes were summarized in Table 1. Among these probes, 3 targeted two genes, and 2 genes were represented by 2 probes simultaneously. As compared to patients with a favorable chemo-sensitivity score, those with an unfavorable score had a shorter OS in both samples with U133 AB (*p*=0.006, HR=1.739, 95% CI: 1.174-2.575, Figure 3A) and U133 plus data (*p*=0.003, HR=2.456, 95% CI: 1.358-4.441, Figure 3B). As age was significantly associated with OS in both U133 AB and U133 plus samples in univariate analysis, we performed further multivariate analysis adjusted by age. The results showed that the influence of chemo-sensitivity score on OS was more notable (*p*=5.13 × 10^−4^, HR=2.040, 95% CI: 1.364-3.051 for U133AB sample; *p*=0.002, HR=2.528, 95% CI: 1.393-4.591 in U133 plus sample).

### Validation of the association between 42-probe-based chemo-sensitivity score and survival in TCGA CN-AML patients

**Figure 4:**
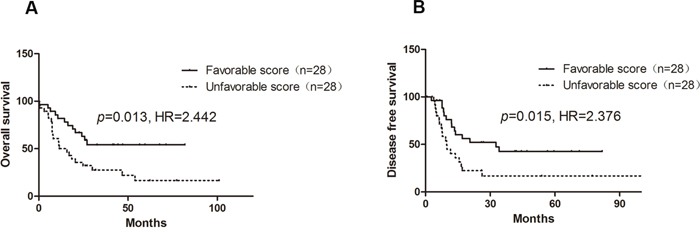
Influence of chemo-sensitivity score on OS and DFS of CN-AML in TCGA AML dataset

TCGA AML dataset was downloaded and association between chemo-sensitivity score based on the 42 probes and OS in 56 *de novo* CN-AML patients was also assessed. Patients with an unfavorable score had a shorter OS (*p*=0.013, HR=2.442, 95% CI: 1.205-4.950, Figure 4A). However, age was not associated with OS in TCGA CN-AML patients (*p*=0.367). As disease-free survival (DFS) data was also available for the TCGA AML dataset. Our results showed that patients with an unfavorable score had significantly shorter DFS as compared with patients with a favorable chemo-sensitivity (*p*=0.015, HR=2.376, 95% CI: 1.181-4.779, Figure 4B).

To confirm whether chemo-sensitivity score was an independent risk factor for OS in TCGA CN-AML patients, association between the score and some known risk factors for AML outcome, including cytogenetic risk, *NPM1* mutations, *FLT3-IDT* mutations, *CEBPA* mutations, *IDH* mutations, and *DMNT3A* R882 mutations were analyzed. However, no significant association was observed for any of the somatic mutations (Table 3).

**Table 3:** Comparison of chemo-sensitivity score between genotypes of well-known somatic mutations affecting AML outcome

Mutation	Wild type		Mutation		*p* value
	Score (Mean±SD)	N	Score (Mean±SD)	N	
*CEBPA*	0.612±3.561	48	−2.557±3.428	5	0.063
*DNMT3A R882*	−0.109±3.396	42	1.926±4.239	11	0.099
*FLT3-IDT*	−0.185±3.705	36	1.369±3.355	17	0.148
*IDH*	0.780±3.849	39	−0.986±2.683	14	0.120
*NPM1*	−0.022±3.925	21	0.533±3.485	32	0.592
*RUNX1*	0.295±3.740	49	0.535±2.373	4	0.901
*WT1*	0.354±3.762	48	−0.076±2.379	5	0.804

## DISCUSSION

In this study, we derived a gene signature including 42 probes which mapped to 43 annotated genes from GDSC blood cell lines and the GEO GSE12417 dataset. The chemo-sensitivity score calculated based on the expression level of the 42 probes was significantly associated with OS in GSE12417 CN-AML patients and replicated in the TCGA AML dataset. Multivariate analysis showed that the chemo-sensitivity score might be an independent risk factor for AML outcome. Our results indicated that chemo-sensitivity score might be used for predicting prognosis of AML patients after cytarabine based chemotherapy.

Cytarabine is a key drug used for the induction therapy of AML. Evidence has shown that AML patients who achieved CR had longer OS than non-CR patients [18–20], and chemo-resistance is the main reason for treatment failure in AML [25–27]. Hence, factors affect IC_50_ of cytarabine may also influence prognosis of AML. Our findings presently confirmed the conjecture. In this study, we obtained a gene signature related to both IC_50_ values of cytarabine in blood cancer lines and AML OS accepted induction therapy based on cytarabine. According to the chemo-sensitivity score calculated by the expression level of the gene signature, AML patients could be divided into favorable and unfavorable groups that have different OS and DFS. These results indicated that identification of factors related to cytarabine response on cancer cell lines maybe a viable strategy for finding potential factors affecting AML survival.

For the 44 genes involved in the signature, results of pathway analysis by using the David database showed that 4 GO terms were significantly correlated with cellular component of mitochondrion after multiple testing corrections. Mitochondria is a key organelles in human cells participating in cell apoptosis [28, 29], and it also plays important roles in AML progress and chemosensitivity [30]. Energy is greatly demanded in cancer cells, therefore, more adenosine triphosphate (ATP) synthesized by mitochondrion is required. Using AML cell lines, Vo *et al.* demonstrated that increasing mitochondrial priming enhances chemo-sensitivity, and selection for reduced mitochondrial priming in relapsed AML may be an important determinant for the chemo-resistant phenotype [31]. Xiao *et al* found that mitochondrial ATPsyn-b played an important role in multidrug resistance in AML [32]. Wang *et al* observed that arsenic trioxide (ATO), used for the treatment of acute promyelocytic leukemia (APL), could induce apoptosis at therapeutic concentrations (1–2mM) through the mitochondrial pathway in APL NB4 cells [33].

There is also study shows that AML blast cells have higher copy number of mtDNA and consume more oxygen [34]. When AML cells were treated by tigecycline to inhibit mitochondrial translation, both mitochondrial translated proteins level and oxygen consumption were reduced with subsequent cell death [35]. Moreover, activation of the molecular cascade of apoptosis through mitochondrial is supposed to be an important mechanism by which cytarabine kills cancer cells [35]. Previous research reported that cytarabine could induce the release of reduced form of cytochrome c from mitochondrial into the cytosol and initiated caspase-3 activity, which could prompt apoptotic program and result in cells death in AML cells [36, 37].

We further compared our results with previous studies that focused on the effect of gene expression in cytarabine response or leukemia treatment outcome. In a whole genome expression association (WES) study that focused on cytarabine sensitive and resistant murine cell lines, *SLC14A1* was found to be upregulated in cytarabine-resistant cells [38]. Interestingly, *SLC14A1* is also included in our gene signature, but in an opposite direction. In our results, *SLC14A1* high expression correlated with low IC_50_ values in blood cancer cell lines and acted as a good prognostic predictor in AML patients for both GSE12417 and TCGA CN-AML datasets. We further queried this gene in GEO database, and found that there were remarkable inter-individual variations in the expression of *SLC14A1* in AML patients (Supplementary Figure S1). In the cell line WES study, both B117P and B140P cell lines demonstrated low *SLC14A1* expression and could not reflect the expression distribution of *SLC14A1* in AML patients [38]. This may therefore lead to the controversial findings. Further mechanism study is needed to confirm whether *SLC14A1* expression increases cytarabine response in AML. Another gene, named *TAL1*, was reported to be associated with outcome of T-cell acute lymphoblastic leukemia [39]. As in our results, high *TAL1* expression predicted better prognosis. Three probes in *TPD52* were included in our gene signature. Previous study found that high *TPD52* expression was correlated with worse outcome in infant ALL, and this was consistent with our results [40]. Moreover, *UCK1, SLC25A38, VDAC1* in our signature have been reported to be involved in proliferation or apoptosis of leukemia cell lines [41–43]. We also compared our signature with other prognostic signatures for AML. All the genes but *SLC25A37* in our signature were reported for the first time [4, 21, 22, 44, 45]. These indicate that our research tactics might help researchers to find new functionally relevant genes involved in AML progression or cytarabine efficacy.

There are also some limitations for this study. First of all, the data used in this study is obtained from public database, therefore information for some common AML somatic mutations and other known factors affecting AML drug response were not available for constructing prediction model, which may result in uncertainty of whether the chemo-sensitivity score was an independent factor. Integration of gene expression data and somatic mutation data is supposed to build a more precision prediction model. Secondly, only 17 AML cell lines are deposited in GDSC database, and in order to gain better statistic power of linear regression analysis, all the blood cancer cell lines were used to screen the probes that were associated with IC_50_ of cytarabine. Therefore, this process might cause false positive results due to specificity of blood cancers. Finally, we failed to validate our prediction model in prospective study. Therefore, further studies are required to validate the clinic significance of the model.

In conclusion, we identified a novel gene set that could affect both the cytarabine sensitivity in blood cell lines and OS of AML patients underwent cytarabine therapy. The prediction model based on the gene set could predict prognosis of CN-AML. Most genes included in the set are correlated with mitochondrial, which hints that mitochondrial might be important in cytarabine response and AML outcomes. Our study proposed a new way to identify factors impacting AML prognosis, and this would provide some scientific basis for elucidating the individual difference in outcomes of AML and realizing personalized medicine according to gene expression pattern of AML.

## MATERIALS AND METHODS

### Samples

#### GDSC cell lines

The open accessed database GDSC (http://www.cancerrxgene.org/) includes more than 800 cancer cell lines with whole genome mRNA expression, mutation and copy number variation information. Based on GDSC release 5.0, 96 blood cancer cell lines with both natural log transformed IC_50_ values of cytarabine and normalized whole genome mRNA expression levels were selected in our study (Supplementary Table S1). The whole genome mRNA expression level of the cell lines were detected by Affymetrix GeneChip Human Genome U133A Array. The normalized method was Affymetrix Micro Array Suite 5.0 algorithm. Details for data processing method are described elsewhere [15]. As in some cell lines, mRNA expression levels were tested by more than two microarrays, and mRNA expression level results were randomly selected from one of the arrays during data analysis.

#### GEO initial sample

The dataset GSE12417 included 242 cytogenetically normal AML patients with whole genome mRNA expression data and clinical prognostic information is available from the GEO database [21]. Three types of arrays were used to detect the whole genome mRNA expression levels: Affymetrix GeneChip Human Genome U133A and U133B arrays (U133 AB sample) for 163 patients, and Affymetrix GeneChip Human Genome U133 plus 2.0 array (U133 plus sample) for the other 79 patients. The MAS5 algorithm was used to normalize the expression data.

#### TCGA validation sample

The TCGA AML dataset includes 200 *de novo* patients [46]. As the TCGA dataset is very complex, only patients with cytogenetically normal, non-M3 subtype, induction chemotherapy based on cytarabine, and percentage of blast cells more than 50% were selected in our analysis. Gene expression level of this dataset was detected by Affymetrix GeneChip Human Genome U133 plus 2.0 array and normalized by MAS5 algorithm. The clinical information of TCGA AML dataset was downloaded from cBioPortal, a well-known tool used to extract and manage data from TCGA database [47].

### Statistical analysis

Linear-regression analysis was performed to identify probes correlated with cytarabine IC_50_ in 96 blood cancer cell lines. Probes with *P*<0.05 were then selected for analysis in the GSE12417 dataset. Multivariate cox regression analysis (including age) was conducted to confirm whether the probes affect OS of CN-AML patients. Probes exhibited significance (*p*<0.05) in both the linear-regression analysis and the cox regression analysis in the same direction were screened. For example, if high expression of a probe was associated with high cytarabine IC_50_ value, the high expression of this probe must correlate with poor AML prognosis.

To obtain the chemo-sensitivity score for each patients, the expression values of the selected probes were standardized transformed (centered to a mean of 0 and then scaled the SD). Then, the univariate cox scores (which indicated the correlation between probe expression levels and OS of the AML patients in GSE12417) of the selected probes were acquired and used to calculate combined cox score for each probe by the following formula: *CCSi*=α_*i*1_×β_1_+α_*i*2_×β_2_, where *CCSi* was the combined cox score of probe *i*. Because GSE12417 included two samples, α_*i*1_ and α_*i*2_ represented the univariate cox score for U133 AB sample and U133 plus sample, respectively; while β_1_ and β_2_ were the proportion of U133 AB and U133 plus samples in GSE12417, respectively. To get the chemo-sensitivity score of each sample, the following formula was used: $\text{CSj}=\sum_{t=1}^{n}\text{ccsi}\times \gamma^{\text{ij}}$, where *CSj* was the chemo-sensitivity score of patient *j*, *n* was the number of selected probes, and *γ*^*ij*^ was the expression level of probe *i* in patient *j*.

**Figure 5:**
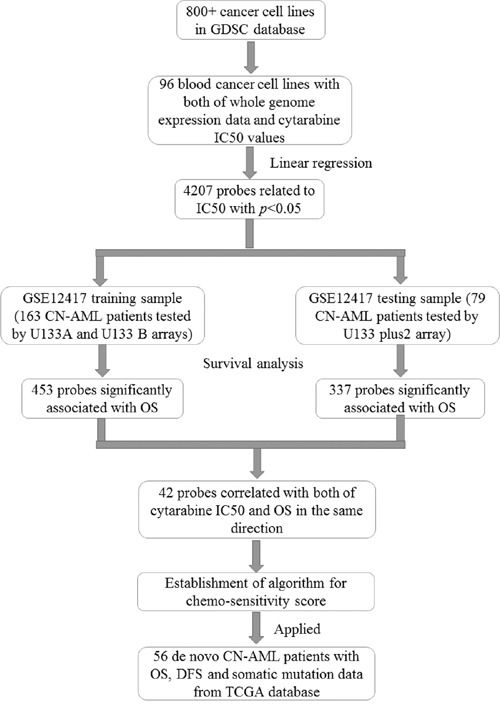
Methods flow chart of analysis process

To obtain the Kaplan-Meier plots of chemo-sensitivity score, patients were divided into favorable and unfavorable groups based on their chemo-sensitivity scores. The median was used as cut-point. Patients with chemo-sensitivity scores equal to or higher than the median were classified as unfavorable, the others were classified as favorable. Heatmap.2 command in gplots package was utilized for the cluster analysis. Gene Ontology (GO) and pathway analysis were conducted by the tool David 6.7 (https://david.ncifcrf.gov/). The GO analysis was performed based on biological process (BP), cellular component (CC) and molecular function (MF). The Kaplan-Meier plots were generated by Graphpad 5.0. All of the data arrangement and statistical analysis were conducted by using R (version 3.1.2). Methods flow chart of analysis process was shown in Figure 5.

## SUPPLEMENTARY FIGURE AND TABLES






