# Supplementary material for: Association of a cytarabine chemosensitivity related gene expression signature with survival in cytogenetically normal acute myeloid leukemia
Source: Oncotarget. 2016 Nov 26;8(1):1529–40. doi: 10.18632/oncotarget.13650 (PMC5352074; doi:10.18632/oncotarget.13650)
Supplement: Supplementary file 1 [file oncotarget-08-1529-s001.pdf]

Association of a cytarabine chemosensitivity related gene expression signature with survival in cytogenetically normal acute myeloid leukemia

SUPPLEMENTARY FIGURE AND TABLES

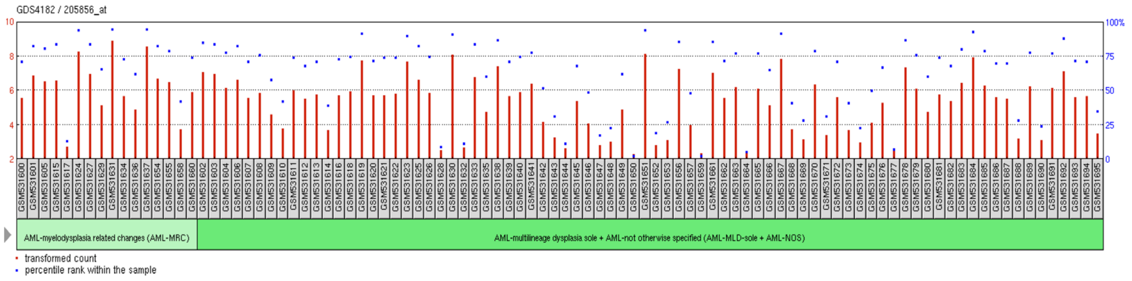

Supplementary Figure S1: Distribution of SLC14A1 expression in AML patients (from GEO GDS4182).

Supplementary Table S1: Annotation of 96 blood cancer cell lines

See Supplementary File 1

Supplementary Table S2: Pathway analysis results based on 4207 probes

See Supplementary File 2
