# Supplementary material for: Association of a cytarabine chemosensitivity related gene expression signature with survival in cytogenetically normal acute myeloid leukemia
Source: Oncotarget. 2016 Nov 26;8(1):1529–40. doi: 10.18632/oncotarget.13650 (PMC5352074; doi:10.18632/oncotarget.13650)
Supplement: Supplementary file 2 [file oncotarget-08-1529-s002.doc]

Table S1 Annotation of 96 blood cancer cell lines

| Cell Line Name | Tissue | IC 50 |
| --- | --- | --- |
| HL-60 | Acute myeloid leukaemia | 1.015639 |
| CESS | Acute myeloid leukaemia | 2.366503 |
| OCI-AML2 | Acute myeloid leukaemia | 1.572669 |
| GDM-1 | Acute myeloid leukaemia | 4.585041 |
| HEL | Acute myeloid leukaemia | -3.88643 |
| KASUMI-1 | Acute myeloid leukaemia | -0.14176 |
| KMOE-2 | Acute myeloid leukaemia | 1.838749 |
| ML-2 | Acute myeloid leukaemia | -1.00229 |
| MONO-MAC-6 | Acute myeloid leukaemia | 0.277365 |
| NKM-1 | Acute myeloid leukaemia | -0.50158 |
| NOMO-1 | Acute myeloid leukaemia | 0.879477 |
| P31-FUJ | Acute myeloid leukaemia | 5.644866 |
| THP-1 | Acute myeloid leukaemia | 5.205835 |
| QIMR-WIL | Acute myeloid leukaemia | 1.892821 |
| CMK | Acute myeloid leukaemia | 0.32922 |
| CTV-1 | Acute myeloid leukaemia | -3.73868 |
| KY821 | Acute myeloid leukaemia | 0.858765 |
| KARPAS-299 | Anaplastic large cell lymphoma | 0.54097 |
| BALL-1 | B cell leukemia | -2.7993 |
| MHH-CALL-2 | B cell leukemia | 0.910539 |
| MHH-PREB-1 | B cell leukemia | -1.64238 |
| MN-60 | B cell leukemia | 3.31683 |
| NALM-6 | B cell leukemia | -1.49809 |
| REH | B cell leukemia | -2.49073 |
| U-698-M | B cell leukemia | 0.618402 |
| RL | B cell lymphoma | 1.078765 |
| BC-1 | B cell lymphoma | 4.563764 |
| A4-Fuk | B cell lymphoma | -0.1789 |
| A3-KAW | B cell lymphoma | 1.042838 |
| CTB-1 | B cell lymphoma | -2.31361 |
| CRO-AP2 | B cell lymphoma | -0.57824 |
| DB | B cell lymphoma | -0.9426 |
| DOHH-2 | B cell lymphoma | 0.321066 |
| HT | B cell lymphoma | 2.549258 |
| KARPAS-422 | B cell lymphoma | 3.419733 |
| TUR | B cell lymphoma | -3.36351 |
| CA46 | Burkitt lymphoma | 3.845369 |
| BL-41 | Burkitt lymphoma | -1.16128 |
| ST486 | Burkitt lymphoma | 1.502442 |
| Daudi | Burkitt lymphoma | 4.424954 |
| DG-75 | Burkitt lymphoma | 0.093137 |
| EB2 | Burkitt lymphoma | 5.467646 |
| EB-3 | Burkitt lymphoma | 4.590446 |
| JiyoyeP-2003 | Burkitt lymphoma | 4.491708 |
| Raji | Burkitt lymphoma | 1.195797 |
| Ramos-2G6-4C10 | Burkitt lymphoma | 1.017862 |
| BV-173 | Chronic myeloid leukaemia | -4.30232 |
| EM-2 | Chronic myeloid leukaemia | -2.23758 |
| LAMA-84 | Chronic myeloid leukaemia | -2.76503 |
| MEG-01 | Chronic myeloid leukaemia | -0.64474 |
| RPMI-8866 | Chronic myeloid leukaemia | -0.11059 |
| EoL-1-cell | Haematopoietic neoplasm other | -0.7842 |
| SKM-1 | Haematopoietic neoplasm other | 1.152746 |
| HC-1 | Hairy cell leukaemia | 4.621674 |
| HDLM-2 | Hodgkin lymphoma | 2.32116 |
| HD-MY-Z | Hodgkin lymphoma | -0.29476 |
| L-428 | Hodgkin lymphoma | 0.826095 |
| L-540 | Hodgkin lymphoma | 1.692527 |
| RPMI-6666 | Hodgkin lymphoma | 1.736239 |
| KM-H2 | Hodgkin lymphoma | 5.142553 |
| RS4-11 | Leukemia | -0.96496 |
| CCRF-CEM | Lymphoblastic leukemia | -3.9803 |
| ALL-PO | Lymphoblastic leukemia | -0.75827 |
| HAL-01 | Lymphoblastic leukemia | -3.38489 |
| MOLT-4 | Lymphoblastic leukemia | -2.37515 |
| BE-13 | Lymphoblastic leukemia | -0.55152 |
| 697 | Lymphoblastic leukemia | -5.23818 |
| GR-ST | Lymphoblastic leukemia | -2.21748 |
| KARPAS-45 | Lymphoblastic leukemia | 2.88941 |
| KE-37 | Lymphoblastic leukemia | -5.32226 |
| LC4-1 | Lymphoblastic leukemia | 2.106036 |
| P30-OHK | Lymphoblastic leukemia | -2.99415 |
| ATN-1 | Lymphoblastic T cell leukaemia | -1.94146 |
| J-RT3-T3-5 | Lymphoblastic T cell leukaemia | -3.3379 |
| LOUCY | Lymphoblastic T cell leukaemia | 0.450336 |
| MOLT-13 | Lymphoblastic T cell leukaemia | -1.31721 |
| MOLT-16 | Lymphoblastic T cell leukaemia | -3.42808 |
| P12-ICHIKAWA | Lymphoblastic T cell leukaemia | -1.80868 |
| PF-382 | Lymphoblastic T cell leukaemia | -1.02984 |
| RPMI-8402 | Lymphoblastic T cell leukaemia | -2.17607 |
| SUP-T1 | Lymphoblastic T cell leukaemia | 1.137216 |
| SR | Lymphoid neoplasm other | -0.40463 |
| DEL | Lymphoid neoplasm other | 3.375236 |
| H9 | Lymphoid neoplasm other | -1.39327 |
| JVM-2 | Lymphoid neoplasm other | 1.374723 |
| JVM-3 | Lymphoid neoplasm other | 0.238527 |
| WSU-NHL | Lymphoid neoplasm other | -2.09057 |
| SK-MM-2 | Myeloma | 4.861502 |
| U-266 | Myeloma | 2.743255 |
| L-363 | Myeloma | 1.135533 |
| RPMI-8226 | Myeloma | 3.214298 |
| ARH-77 | Myeloma | 1.9405 |
| LP-1 | Myeloma | 4.59891 |
| OPM-2 | Myeloma | 4.80532 |
| IM-9 | Myeloma | 4.624077 |
| HH | T cell leukemia | 1.40307 |
