# Supplementary material for: Association of a cytarabine chemosensitivity related gene expression signature with survival in cytogenetically normal acute myeloid leukemia
Source: Oncotarget. 2016 Nov 26;8(1):1529–40. doi: 10.18632/oncotarget.13650 (PMC5352074; doi:10.18632/oncotarget.13650)
Supplement: Supplementary file 3 [file oncotarget-08-1529-s003.doc]

**Pathway analysis results based on 4207 probes**

| **Category** | **Term** | **P Value** | **Bonferroni** | **FDR** |
| --- | --- | --- | --- | --- |
| GOTERM_CC_FAT | GO:0070013~intracellular organelle lumen | 4.44E-31 | 3.25E-28 | 6.73E-28 |
| GOTERM_CC_FAT | GO:0031974~membrane-enclosed lumen | 2.15E-29 | 1.58E-26 | 3.27E-26 |
| GOTERM_CC_FAT | GO:0043233~organelle lumen | 2.54E-29 | 1.86E-26 | 3.85E-26 |
| GOTERM_CC_FAT | GO:0005654~nucleoplasm | 1.79E-25 | 1.31E-22 | 2.71E-22 |
| GOTERM_CC_FAT | GO:0031981~nuclear lumen | 8.62E-25 | 6.31E-22 | 1.31E-21 |
| GOTERM_MF_FAT | GO:0000166~nucleotide binding | 3.45E-16 | 5.83E-13 | 5.66E-13 |
| GOTERM_CC_FAT | GO:0005829~cytosol | 1.64E-13 | 1.20E-10 | 2.49E-10 |
| GOTERM_CC_FAT | GO:0044451~nucleoplasm part | 5.51E-13 | 4.03E-10 | 8.35E-10 |
| GOTERM_BP_FAT | GO:0006397~mRNA processing | 3.51E-12 | 1.67E-08 | 6.64E-09 |
| GOTERM_BP_FAT | GO:0046907~intracellular transport | 9.27E-12 | 4.40E-08 | 1.75E-08 |
| GOTERM_CC_FAT | GO:0043232~intracellular non-membrane-bounded organelle | 1.44E-11 | 1.06E-08 | 2.19E-08 |
| GOTERM_CC_FAT | GO:0043228~non-membrane-bounded organelle | 1.44E-11 | 1.06E-08 | 2.19E-08 |
| GOTERM_MF_FAT | GO:0003723~RNA binding | 1.63E-11 | 2.84E-08 | 2.75E-08 |
| GOTERM_MF_FAT | GO:0005524~ATP binding | 1.73E-11 | 3.02E-08 | 2.92E-08 |
| GOTERM_BP_FAT | GO:0006396~RNA processing | 2.74E-11 | 1.30E-07 | 5.18E-08 |
| GOTERM_MF_FAT | GO:0032559~adenyl ribonucleotide binding | 4.63E-11 | 8.09E-08 | 7.83E-08 |
| GOTERM_BP_FAT | GO:0008380~RNA splicing | 6.12E-11 | 2.90E-07 | 1.16E-07 |
| GOTERM_BP_FAT | GO:0015031~protein transport | 8.04E-11 | 3.82E-07 | 1.52E-07 |
| GOTERM_BP_FAT | GO:0045184~establishment of protein localization | 1.14E-10 | 5.42E-07 | 2.16E-07 |
| GOTERM_MF_FAT | GO:0032553~ribonucleotide binding | 1.45E-10 | 2.53E-07 | 2.45E-07 |
| GOTERM_MF_FAT | GO:0032555~purine ribonucleotide binding | 1.45E-10 | 2.53E-07 | 2.45E-07 |
| GOTERM_BP_FAT | GO:0046649~lymphocyte activation | 1.71E-10 | 8.13E-07 | 3.24E-07 |
| GOTERM_BP_FAT | GO:0008104~protein localization | 3.08E-10 | 1.46E-06 | 5.84E-07 |
| GOTERM_MF_FAT | GO:0001883~purine nucleoside binding | 3.56E-10 | 6.22E-07 | 6.02E-07 |
| GOTERM_MF_FAT | GO:0030554~adenyl nucleotide binding | 3.56E-10 | 6.22E-07 | 6.02E-07 |
| GOTERM_MF_FAT | GO:0001882~nucleoside binding | 5.84E-10 | 1.02E-06 | 9.88E-07 |
| GOTERM_MF_FAT | GO:0017076~purine nucleotide binding | 6.48E-10 | 1.13E-06 | 1.10E-06 |
| GOTERM_BP_FAT | GO:0016071~mRNA metabolic process | 6.85E-10 | 3.25E-06 | 1.30E-06 |
| GOTERM_BP_FAT | GO:0045321~leukocyte activation | 7.95E-10 | 3.77E-06 | 1.50E-06 |
| GOTERM_BP_FAT | GO:0000398~nuclear mRNA splicing, via spliceosome | 1.52E-09 | 7.20E-06 | 2.87E-06 |
| GOTERM_BP_FAT | GO:0000377~RNA splicing, via transesterification reactions with bulged adenosine as nucleophile | 1.52E-09 | 7.20E-06 | 2.87E-06 |
| GOTERM_BP_FAT | GO:0000375~RNA splicing, via transesterification reactions | 1.52E-09 | 7.20E-06 | 2.87E-06 |
| GOTERM_BP_FAT | GO:0042110~T cell activation | 1.90E-09 | 9.02E-06 | 3.60E-06 |
| GOTERM_BP_FAT | GO:0043067~regulation of programmed cell death | 2.60E-09 | 1.23E-05 | 4.91E-06 |
| GOTERM_BP_FAT | GO:0042981~regulation of apoptosis | 2.86E-09 | 1.36E-05 | 5.41E-06 |
| GOTERM_BP_FAT | GO:0010941~regulation of cell death | 3.69E-09 | 1.75E-05 | 6.99E-06 |
| GOTERM_BP_FAT | GO:0043933~macromolecular complex subunit organization | 6.61E-09 | 3.13E-05 | 1.25E-05 |
| GOTERM_BP_FAT | GO:0001775~cell activation | 7.29E-09 | 3.46E-05 | 1.38E-05 |
| GOTERM_CC_FAT | GO:0016604~nuclear body | 2.46E-08 | 1.80E-05 | 3.73E-05 |
| GOTERM_BP_FAT | GO:0065003~macromolecular complex assembly | 4.67E-08 | 2.21E-04 | 8.83E-05 |
| GOTERM_BP_FAT | GO:0016310~phosphorylation | 9.10E-08 | 4.31E-04 | 1.72E-04 |
| GOTERM_CC_FAT | GO:0005730~nucleolus | 9.65E-08 | 7.07E-05 | 1.46E-04 |
| GOTERM_CC_FAT | GO:0031090~organelle membrane | 9.94E-08 | 7.28E-05 | 1.51E-04 |
| GOTERM_BP_FAT | GO:0006793~phosphorus metabolic process | 1.53E-07 | 7.26E-04 | 2.89E-04 |
| GOTERM_BP_FAT | GO:0006796~phosphate metabolic process | 1.53E-07 | 7.26E-04 | 2.89E-04 |
| GOTERM_BP_FAT | GO:0007049~cell cycle | 1.97E-07 | 9.35E-04 | 3.73E-04 |
| GOTERM_MF_FAT | GO:0004672~protein kinase activity | 2.06E-07 | 3.60E-04 | 3.48E-04 |
| GOTERM_BP_FAT | GO:0016265~death | 2.37E-07 | 0.001121651 | 4.48E-04 |
| GOTERM_MF_FAT | GO:0004674~protein serine/threonine kinase activity | 2.47E-07 | 4.32E-04 | 4.18E-04 |
| GOTERM_CC_FAT | GO:0005794~Golgi apparatus | 3.29E-07 | 2.41E-04 | 4.99E-04 |
| GOTERM_BP_FAT | GO:0008219~cell death | 3.59E-07 | 0.001700132 | 6.79E-04 |
| GOTERM_BP_FAT | GO:0006259~DNA metabolic process | 5.22E-07 | 0.002472379 | 9.87E-04 |
| GOTERM_CC_FAT | GO:0012505~endomembrane system | 5.75E-07 | 4.21E-04 | 8.72E-04 |
| GOTERM_MF_FAT | GO:0008134~transcription factor binding | 6.82E-07 | 0.001192516 | 0.001155 |
| GOTERM_BP_FAT | GO:0006468~protein amino acid phosphorylation | 6.88E-07 | 0.00325887 | 0.001302 |
| GOTERM_BP_FAT | GO:0030098~lymphocyte differentiation | 7.47E-07 | 0.003538791 | 0.001414 |
| GOTERM_MF_FAT | GO:0016563~transcription activator activity | 1.02E-06 | 0.001787912 | 0.001732 |
| GOTERM_CC_FAT | GO:0016607~nuclear speck | 1.26E-06 | 9.22E-04 | 0.001911 |
| GOTERM_BP_FAT | GO:0006281~DNA repair | 1.40E-06 | 0.006609224 | 0.002645 |
| GOTERM_BP_FAT | GO:0008283~cell proliferation | 1.41E-06 | 0.006676708 | 0.002672 |
| GOTERM_BP_FAT | GO:0006974~response to DNA damage stimulus | 1.66E-06 | 0.007836033 | 0.003138 |
| GOTERM_BP_FAT | GO:0043068~positive regulation of programmed cell death | 1.67E-06 | 0.007900473 | 0.003164 |
| GOTERM_BP_FAT | GO:0043065~positive regulation of apoptosis | 1.98E-06 | 0.009334202 | 0.00374 |
| GOTERM_BP_FAT | GO:0010942~positive regulation of cell death | 2.15E-06 | 0.010156269 | 0.004072 |
| GOTERM_CC_FAT | GO:0005694~chromosome | 3.28E-06 | 0.002399522 | 0.004979 |
| GOTERM_CC_FAT | GO:0044431~Golgi apparatus part | 3.71E-06 | 0.002710408 | 0.005625 |
| GOTERM_BP_FAT | GO:0012501~programmed cell death | 5.52E-06 | 0.025843621 | 0.010443 |
| GOTERM_BP_FAT | GO:0010605~negative regulation of macromolecule metabolic process | 5.78E-06 | 0.027060399 | 0.010941 |
| GOTERM_CC_FAT | GO:0005768~endosome | 5.93E-06 | 0.004331175 | 0.008996 |
| GOTERM_CC_FAT | GO:0000792~heterochromatin | 5.99E-06 | 0.004375891 | 0.009089 |
| GOTERM_BP_FAT | GO:0006461~protein complex assembly | 6.04E-06 | 0.028261185 | 0.011434 |
| GOTERM_BP_FAT | GO:0070271~protein complex biogenesis | 6.04E-06 | 0.028261185 | 0.011434 |
| GOTERM_BP_FAT | GO:0006367~transcription initiation from RNA polymerase II promoter | 6.16E-06 | 0.028805622 | 0.011657 |
| GOTERM_BP_FAT | GO:0006352~transcription initiation | 9.02E-06 | 0.041910629 | 0.017075 |
| GOTERM_BP_FAT | GO:0006886~intracellular protein transport | 1.04E-05 | 0.047967099 | 0.019604 |
| GOTERM_BP_FAT | GO:0070727~cellular macromolecule localization | 1.16E-05 | 0.053488604 | 0.021924 |
| GOTERM_BP_FAT | GO:0022402~cell cycle process | 1.20E-05 | 0.055314037 | 0.022693 |
| GOTERM_BP_FAT | GO:0006917~induction of apoptosis | 1.31E-05 | 0.060173329 | 0.02475 |
| GOTERM_BP_FAT | GO:0006915~apoptosis | 1.31E-05 | 0.060198049 | 0.02476 |
| GOTERM_BP_FAT | GO:0033554~cellular response to stress | 1.33E-05 | 0.061149569 | 0.025164 |
| GOTERM_BP_FAT | GO:0034613~cellular protein localization | 1.36E-05 | 0.062599306 | 0.02578 |
| GOTERM_BP_FAT | GO:0010627~regulation of protein kinase cascade | 1.42E-05 | 0.064954429 | 0.026784 |
| GOTERM_CC_FAT | GO:0005739~mitochondrion | 1.47E-05 | 0.010678174 | 0.022247 |
| GOTERM_BP_FAT | GO:0012502~induction of programmed cell death | 1.50E-05 | 0.068573386 | 0.02833 |
| GOTERM_MF_FAT | GO:0003725~double-stranded RNA binding | 1.86E-05 | 0.032067845 | 0.031533 |
| GOTERM_MF_FAT | GO:0003682~chromatin binding | 2.11E-05 | 0.036305142 | 0.035777 |
| GOTERM_BP_FAT | GO:0002521~leukocyte differentiation | 2.25E-05 | 0.101176842 | 0.042536 |
| GOTERM_BP_FAT | GO:0030217~T cell differentiation | 2.28E-05 | 0.102442674 | 0.043098 |
| GOTERM_CC_FAT | GO:0044427~chromosomal part | 2.67E-05 | 0.019319857 | 0.040424 |
| GOTERM_BP_FAT | GO:0007242~intracellular signaling cascade | 2.80E-05 | 0.124400588 | 0.052973 |
| GOTERM_BP_FAT | GO:0051028~mRNA transport | 2.86E-05 | 0.126935775 | 0.054128 |
| GOTERM_BP_FAT | GO:0006351~transcription, DNA-dependent | 2.99E-05 | 0.132208236 | 0.056543 |
| GOTERM_BP_FAT | GO:0034621~cellular macromolecular complex subunit organization | 3.16E-05 | 0.139208364 | 0.059772 |
| GOTERM_BP_FAT | GO:0002520~immune system development | 3.43E-05 | 0.150158547 | 0.064875 |
| GOTERM_CC_FAT | GO:0044432~endoplasmic reticulum part | 3.52E-05 | 0.025424612 | 0.05336 |
| GOTERM_CC_FAT | GO:0005769~early endosome | 3.57E-05 | 0.02579848 | 0.054155 |
| GOTERM_MF_FAT | GO:0008022~protein C-terminus binding | 3.87E-05 | 0.065467706 | 0.065496 |
| GOTERM_BP_FAT | GO:0048534~hemopoietic or lymphoid organ development | 3.91E-05 | 0.169144913 | 0.073881 |
| GOTERM_BP_FAT | GO:0048002~antigen processing and presentation of peptide antigen | 4.47E-05 | 0.191110534 | 0.084559 |
| GOTERM_BP_FAT | GO:0030097~hemopoiesis | 4.67E-05 | 0.198600428 | 0.088266 |
| GOTERM_BP_FAT | GO:0032774~RNA biosynthetic process | 5.10E-05 | 0.214802958 | 0.096405 |
| GOTERM_CC_FAT | GO:0005783~endoplasmic reticulum | 5.23E-05 | 0.037553996 | 0.079299 |
| GOTERM_BP_FAT | GO:0000278~mitotic cell cycle | 5.24E-05 | 0.220180894 | 0.099144 |
| GOTERM_CC_FAT | GO:0030529~ribonucleoprotein complex | 5.37E-05 | 0.038535553 | 0.081412 |
| GOTERM_BP_FAT | GO:0050657~nucleic acid transport | 5.65E-05 | 0.235066371 | 0.106823 |
| GOTERM_BP_FAT | GO:0050658~RNA transport | 5.65E-05 | 0.235066371 | 0.106823 |
| GOTERM_BP_FAT | GO:0051236~establishment of RNA localization | 5.65E-05 | 0.235066371 | 0.106823 |
| GOTERM_BP_FAT | GO:0043066~negative regulation of apoptosis | 6.25E-05 | 0.256783111 | 0.118297 |
| GOTERM_BP_FAT | GO:0006366~transcription from RNA polymerase II promoter | 6.45E-05 | 0.263717139 | 0.122032 |
| GOTERM_BP_FAT | GO:0019882~antigen processing and presentation | 6.97E-05 | 0.281621556 | 0.131838 |
| GOTERM_CC_FAT | GO:0044429~mitochondrial part | 7.05E-05 | 0.050332376 | 0.106974 |
| GOTERM_BP_FAT | GO:0050870~positive regulation of T cell activation | 7.54E-05 | 0.300927175 | 0.142689 |
| GOTERM_CC_FAT | GO:0031967~organelle envelope | 7.60E-05 | 0.054143381 | 0.115299 |
| GOTERM_CC_FAT | GO:0015630~microtubule cytoskeleton | 8.64E-05 | 0.061255082 | 0.130921 |
| GOTERM_CC_FAT | GO:0031975~envelope | 8.83E-05 | 0.062596445 | 0.133881 |
| GOTERM_MF_FAT | GO:0003712~transcription cofactor activity | 9.73E-05 | 0.156509635 | 0.164562 |
| GOTERM_BP_FAT | GO:0043122~regulation of I-kappaB kinase/NF-kappaB cascade | 9.93E-05 | 0.375894305 | 0.187858 |
| GOTERM_CC_FAT | GO:0015629~actin cytoskeleton | 9.98E-05 | 0.07042314 | 0.151233 |
| GOTERM_BP_FAT | GO:0043069~negative regulation of programmed cell death | 1.11E-04 | 0.409144205 | 0.209651 |
| GOTERM_MF_FAT | GO:0003713~transcription coactivator activity | 1.13E-04 | 0.179960955 | 0.191798 |
| GOTERM_BP_FAT | GO:0006403~RNA localization | 1.15E-04 | 0.420558464 | 0.217415 |
| GOTERM_BP_FAT | GO:0016192~vesicle-mediated transport | 1.19E-04 | 0.431010428 | 0.224659 |
| GOTERM_BP_FAT | GO:0051276~chromosome organization | 1.21E-04 | 0.436623895 | 0.228605 |
| GOTERM_BP_FAT | GO:0060548~negative regulation of cell death | 1.24E-04 | 0.444690451 | 0.234344 |
| GOTERM_CC_FAT | GO:0016585~chromatin remodeling complex | 1.37E-04 | 0.095711601 | 0.208293 |
| GOTERM_BP_FAT | GO:0043123~positive regulation of I-kappaB kinase/NF-kappaB cascade | 1.38E-04 | 0.481623852 | 0.261727 |
| GOTERM_CC_FAT | GO:0000785~chromatin | 1.39E-04 | 0.096474507 | 0.210038 |
| GOTERM_BP_FAT | GO:0016568~chromatin modification | 1.45E-04 | 0.497683162 | 0.274245 |
| GOTERM_CC_FAT | GO:0005643~nuclear pore | 1.49E-04 | 0.103086813 | 0.225228 |
| GOTERM_CC_FAT | GO:0005681~spliceosome | 1.59E-04 | 0.109993407 | 0.241212 |
| GOTERM_MF_FAT | GO:0003697~single-stranded DNA binding | 1.62E-04 | 0.247165387 | 0.274344 |
| GOTERM_CC_FAT | GO:0042825~TAP complex | 1.94E-04 | 0.132692362 | 0.294612 |
| GOTERM_BP_FAT | GO:0006325~chromatin organization | 2.05E-04 | 0.622564248 | 0.387873 |
| GOTERM_BP_FAT | GO:0006376~mRNA splice site selection | 2.54E-04 | 0.70041496 | 0.47961 |
| GOTERM_CC_FAT | GO:0005759~mitochondrial matrix | 2.67E-04 | 0.177269256 | 0.403586 |
| GOTERM_CC_FAT | GO:0031980~mitochondrial lumen | 2.67E-04 | 0.177269256 | 0.403586 |
| GOTERM_BP_FAT | GO:0006916~anti-apoptosis | 2.70E-04 | 0.722640654 | 0.510203 |
| GOTERM_BP_FAT | GO:0008624~induction of apoptosis by extracellular signals | 2.85E-04 | 0.741557411 | 0.538231 |
| GOTERM_BP_FAT | GO:0000245~spliceosome assembly | 3.13E-04 | 0.773283016 | 0.590175 |
| GOTERM_MF_FAT | GO:0016251~general RNA polymerase II transcription factor activity | 3.18E-04 | 0.426998572 | 0.537394 |
| GOTERM_BP_FAT | GO:0002474~antigen processing and presentation of peptide antigen via MHC class I | 3.18E-04 | 0.779331858 | 0.600896 |
| GOTERM_BP_FAT | GO:0046651~lymphocyte proliferation | 3.25E-04 | 0.786359486 | 0.613727 |
| GOTERM_BP_FAT | GO:0015931~nucleobase, nucleoside, nucleotide and nucleic acid transport | 3.47E-04 | 0.807710697 | 0.655458 |
| GOTERM_CC_FAT | GO:0042175~nuclear envelope-endoplasmic reticulum network | 3.48E-04 | 0.225098381 | 0.527138 |
| GOTERM_CC_FAT | GO:0046930~pore complex | 3.73E-04 | 0.238923563 | 0.564244 |
| GOTERM_BP_FAT | GO:0009967~positive regulation of signal transduction | 3.77E-04 | 0.832890426 | 0.711055 |
| GOTERM_BP_FAT | GO:0034622~cellular macromolecular complex assembly | 4.18E-04 | 0.862321069 | 0.787744 |
| GOTERM_MF_FAT | GO:0008092~cytoskeletal protein binding | 4.56E-04 | 0.549285976 | 0.768164 |
| GOTERM_MF_FAT | GO:0019899~enzyme binding | 4.58E-04 | 0.55159805 | 0.773102 |
| GOTERM_CC_FAT | GO:0005856~cytoskeleton | 4.69E-04 | 0.290558769 | 0.708924 |
| GOTERM_BP_FAT | GO:0051172~negative regulation of nitrogen compound metabolic process | 4.93E-04 | 0.903568493 | 0.928555 |
| GOTERM_BP_FAT | GO:0010558~negative regulation of macromolecule biosynthetic process | 5.00E-04 | 0.906681111 | 0.941519 |
| GOTERM_BP_FAT | GO:0031327~negative regulation of cellular biosynthetic process | 5.04E-04 | 0.908563251 | 0.949569 |
| GOTERM_MF_FAT | GO:0004536~deoxyribonuclease activity | 5.14E-04 | 0.593372895 | 0.866954 |
| GOTERM_BP_FAT | GO:0006260~DNA replication | 5.61E-04 | 0.930196246 | 1.056164 |
| GOTERM_CC_FAT | GO:0005802~trans-Golgi network | 5.70E-04 | 0.341364334 | 0.861718 |
| GOTERM_CC_FAT | GO:0005773~vacuole | 6.10E-04 | 0.360321203 | 0.921705 |
| GOTERM_BP_FAT | GO:0009890~negative regulation of biosynthetic process | 6.18E-04 | 0.946889964 | 1.16397 |
| GOTERM_BP_FAT | GO:0070661~leukocyte proliferation | 6.43E-04 | 0.95266615 | 1.209349 |
| GOTERM_BP_FAT | GO:0032943~mononuclear cell proliferation | 6.43E-04 | 0.95266615 | 1.209349 |
| GOTERM_BP_FAT | GO:0045333~cellular respiration | 7.32E-04 | 0.968981566 | 1.375744 |
| GOTERM_BP_FAT | GO:0006310~DNA recombination | 7.41E-04 | 0.970336508 | 1.393312 |
| GOTERM_BP_FAT | GO:0006368~RNA elongation from RNA polymerase II promoter | 7.47E-04 | 0.971121675 | 1.403862 |
| GOTERM_BP_FAT | GO:0010740~positive regulation of protein kinase cascade | 7.86E-04 | 0.976071284 | 1.477771 |
| GOTERM_CC_FAT | GO:0005938~cell cortex | 8.06E-04 | 0.445767346 | 1.21569 |
| GOTERM_MF_FAT | GO:0032395~MHC class II receptor activity | 8.30E-04 | 0.766069147 | 1.395877 |
| GOTERM_BP_FAT | GO:0045934~negative regulation of nucleobase, nucleoside, nucleotide and nucleic acid metabolic process | 8.67E-04 | 0.983660151 | 1.627561 |
| GOTERM_BP_FAT | GO:0044092~negative regulation of molecular function | 8.71E-04 | 0.983955543 | 1.634719 |
| GOTERM_BP_FAT | GO:0006605~protein targeting | 9.00E-04 | 0.986048301 | 1.689538 |
| GOTERM_CC_FAT | GO:0005635~nuclear envelope | 9.13E-04 | 0.487529757 | 1.375951 |
| GOTERM_BP_FAT | GO:0010629~negative regulation of gene expression | 9.38E-04 | 0.988341646 | 1.759928 |
| GOTERM_MF_FAT | GO:0003702~RNA polymerase II transcription factor activity | 9.97E-04 | 0.825390793 | 1.674541 |
| GOTERM_MF_FAT | GO:0019207~kinase regulator activity | 9.97E-04 | 0.825418973 | 1.674695 |
| GOTERM_BP_FAT | GO:0006091~generation of precursor metabolites and energy | 0.001005 | 0.991539367 | 1.88547 |
| GOTERM_BP_FAT | GO:0016481~negative regulation of transcription | 0.00104 | 0.992827994 | 1.950113 |
| GOTERM_BP_FAT | GO:0006357~regulation of transcription from RNA polymerase II promoter | 0.001106 | 0.994766018 | 2.07323 |
| GOTERM_BP_FAT | GO:0022403~cell cycle phase | 0.00113 | 0.99532534 | 2.117362 |
| GOTERM_CC_FAT | GO:0030530~heterogeneous nuclear ribonucleoprotein complex | 0.001132 | 0.56345563 | 1.703168 |
| GOTERM_CC_FAT | GO:0005793~ER-Golgi intermediate compartment | 0.001179 | 0.578259059 | 1.773424 |
| GOTERM_BP_FAT | GO:0051098~regulation of binding | 0.001192 | 0.996517819 | 2.23227 |
| GOTERM_BP_FAT | GO:0042113~B cell activation | 0.001223 | 0.996986792 | 2.288661 |
| GOTERM_CC_FAT | GO:0005626~insoluble fraction | 0.001241 | 0.597096261 | 1.866402 |
| GOTERM_BP_FAT | GO:0051640~organelle localization | 0.001302 | 0.997937589 | 2.436307 |
| GOTERM_CC_FAT | GO:0016591~DNA-directed RNA polymerase II, holoenzyme | 0.001404 | 0.642432643 | 2.108889 |
| GOTERM_CC_FAT | GO:0005789~endoplasmic reticulum membrane | 0.001409 | 0.643816573 | 2.116756 |
| GOTERM_MF_FAT | GO:0030528~transcription regulator activity | 0.00143 | 0.918146553 | 2.392728 |
| GOTERM_CC_FAT | GO:0042611~MHC protein complex | 0.001454 | 0.65538316 | 2.183704 |
| GOTERM_BP_FAT | GO:0050863~regulation of T cell activation | 0.00149 | 0.999155267 | 2.783038 |
| GOTERM_BP_FAT | GO:0009615~response to virus | 0.001536 | 0.999321412 | 2.867922 |
| GOTERM_BP_FAT | GO:0051251~positive regulation of lymphocyte activation | 0.001576 | 0.999437828 | 2.940809 |
| GOTERM_BP_FAT | GO:0006955~immune response | 0.00161 | 0.999521518 | 3.00319 |
| GOTERM_BP_FAT | GO:0050852~T cell receptor signaling pathway | 0.00162 | 0.999543306 | 3.021219 |
| GOTERM_CC_FAT | GO:0042824~MHC class I peptide loading complex | 0.001677 | 0.707267195 | 2.513939 |
| GOTERM_CC_FAT | GO:0005813~centrosome | 0.001688 | 0.709578409 | 2.529953 |
| GOTERM_BP_FAT | GO:0009264~deoxyribonucleotide catabolic process | 0.001696 | 0.999682823 | 3.162127 |
| GOTERM_BP_FAT | GO:0045580~regulation of T cell differentiation | 0.001754 | 0.999759168 | 3.268426 |
| GOTERM_BP_FAT | GO:0006354~RNA elongation | 0.001754 | 0.999759168 | 3.268426 |
| GOTERM_CC_FAT | GO:0005819~spindle | 0.001774 | 0.727353448 | 2.657453 |
| GOTERM_BP_FAT | GO:0022900~electron transport chain | 0.001793 | 0.999800061 | 3.340195 |
| GOTERM_CC_FAT | GO:0005815~microtubule organizing center | 0.001879 | 0.747510218 | 2.81228 |
| GOTERM_CC_FAT | GO:0000267~cell fraction | 0.001906 | 0.752481034 | 2.852322 |
| GOTERM_BP_FAT | GO:0048193~Golgi vesicle transport | 0.001917 | 0.999888918 | 3.566524 |
| GOTERM_BP_FAT | GO:0007243~protein kinase cascade | 0.001969 | 0.999913337 | 3.661957 |
| GOTERM_MF_FAT | GO:0005070~SH3/SH2 adaptor activity | 0.001996 | 0.969644831 | 3.325137 |
| GOTERM_CC_FAT | GO:0016514~SWI/SNF complex | 0.002029 | 0.773877141 | 3.034181 |
| GOTERM_CC_FAT | GO:0070603~SWI/SNF-type complex | 0.002029 | 0.773877141 | 3.034181 |
| GOTERM_CC_FAT | GO:0009898~internal side of plasma membrane | 0.002132 | 0.790274037 | 3.185342 |
| GOTERM_BP_FAT | GO:0009057~macromolecule catabolic process | 0.002191 | 0.999969794 | 4.0661 |
| GOTERM_BP_FAT | GO:0033077~T cell differentiation in the thymus | 0.002199 | 0.999970894 | 4.080301 |
| GOTERM_BP_FAT | GO:0051329~interphase of mitotic cell cycle | 0.002233 | 0.999975274 | 4.142665 |
| GOTERM_MF_FAT | GO:0016564~transcription repressor activity | 0.002235 | 0.980044124 | 3.716714 |
| GOTERM_BP_FAT | GO:0046641~positive regulation of alpha-beta T cell proliferation | 0.002329 | 0.999984305 | 4.316295 |
| GOTERM_BP_FAT | GO:0044265~cellular macromolecule catabolic process | 0.0024 | 0.999988819 | 4.445611 |
| GOTERM_BP_FAT | GO:0006308~DNA catabolic process | 0.002537 | 0.999994184 | 4.694373 |
| GOTERM_BP_FAT | GO:0022613~ribonucleoprotein complex biogenesis | 0.002555 | 0.999994665 | 4.727237 |
| GOTERM_BP_FAT | GO:0006084~acetyl-CoA metabolic process | 0.002741 | 0.999997796 | 5.062601 |
| GOTERM_BP_FAT | GO:0006613~cotranslational protein targeting to membrane | 0.002876 | 0.999998837 | 5.304468 |
| GOTERM_CC_FAT | GO:0005624~membrane fraction | 0.002962 | 0.886019112 | 4.40115 |
| GOTERM_BP_FAT | GO:0010647~positive regulation of cell communication | 0.00299 | 0.999999327 | 5.510427 |
| GOTERM_BP_FAT | GO:0043408~regulation of MAPKKK cascade | 0.003059 | 0.999999515 | 5.634139 |
| GOTERM_CC_FAT | GO:0005764~lysosome | 0.003124 | 0.8987584 | 4.635688 |
| GOTERM_CC_FAT | GO:0000323~lytic vacuole | 0.003124 | 0.8987584 | 4.635688 |
| GOTERM_BP_FAT | GO:0051656~establishment of organelle localization | 0.003332 | 0.999999867 | 6.120496 |
| GOTERM_BP_FAT | GO:0043414~biopolymer methylation | 0.003332 | 0.999999867 | 6.120496 |
| GOTERM_MF_FAT | GO:0008173~RNA methyltransferase activity | 0.003389 | 0.997361844 | 5.58356 |
| GOTERM_CC_FAT | GO:0000118~histone deacetylase complex | 0.003492 | 0.92276767 | 5.169196 |
| GOTERM_CC_FAT | GO:0019898~extrinsic to membrane | 0.003575 | 0.927313066 | 5.288335 |
| GOTERM_CC_FAT | GO:0045120~pronucleus | 0.003659 | 0.931640323 | 5.408739 |
| GOTERM_BP_FAT | GO:0002696~positive regulation of leukocyte activation | 0.00368 | 0.999999975 | 6.739404 |
| GOTERM_BP_FAT | GO:0051325~interphase | 0.00368 | 0.999999975 | 6.739404 |
| GOTERM_CC_FAT | GO:0030863~cortical cytoskeleton | 0.003682 | 0.932792714 | 5.442063 |
| GOTERM_MF_FAT | GO:0019904~protein domain specific binding | 0.003682 | 0.998422745 | 6.052346 |
| GOTERM_MF_FAT | GO:0019900~kinase binding | 0.003711 | 0.998499557 | 6.097721 |
| GOTERM_BP_FAT | GO:0031400~negative regulation of protein modification process | 0.003949 | 0.999999993 | 7.215667 |
| GOTERM_BP_FAT | GO:0043254~regulation of protein complex assembly | 0.003983 | 0.999999994 | 7.275351 |
| GOTERM_BP_FAT | GO:0045637~regulation of myeloid cell differentiation | 0.004086 | 0.999999996 | 7.455453 |
| GOTERM_BP_FAT | GO:0044093~positive regulation of molecular function | 0.004172 | 0.999999998 | 7.607833 |
| GOTERM_MF_FAT | GO:0042288~MHC class I protein binding | 0.004234 | 0.999400959 | 6.928317 |
| GOTERM_BP_FAT | GO:0080135~regulation of cellular response to stress | 0.004426 | 0.999999999 | 8.053236 |
| GOTERM_BP_FAT | GO:0045619~regulation of lymphocyte differentiation | 0.004911 | 1 | 8.89572 |
| GOTERM_CC_FAT | GO:0042598~vesicular fraction | 0.005019 | 0.974847529 | 7.348653 |
| GOTERM_BP_FAT | GO:0006458~'de novo' protein folding | 0.005061 | 1 | 9.155472 |
| GOTERM_BP_FAT | GO:0007005~mitochondrion organization | 0.005211 | 1 | 9.415416 |
| GOTERM_BP_FAT | GO:0006304~DNA modification | 0.005227 | 1 | 9.442931 |
| GOTERM_BP_FAT | GO:0051650~establishment of vesicle localization | 0.005227 | 1 | 9.442931 |
| GOTERM_BP_FAT | GO:0046635~positive regulation of alpha-beta T cell activation | 0.005293 | 1 | 9.555528 |
| GOTERM_BP_FAT | GO:0030183~B cell differentiation | 0.005488 | 1 | 9.891174 |
| GOTERM_MF_FAT | GO:0043021~ribonucleoprotein binding | 0.005506 | 0.999935957 | 8.920222 |
| GOTERM_BP_FAT | GO:0006913~nucleocytoplasmic transport | 0.005679 | 1 | 10.21835 |
| GOTERM_BP_FAT | GO:0043086~negative regulation of catalytic activity | 0.005785 | 1 | 10.39965 |
| GOTERM_BP_FAT | GO:0051648~vesicle localization | 0.00582 | 1 | 10.45897 |
| GOTERM_MF_FAT | GO:0005048~signal sequence binding | 0.005856 | 0.999965421 | 9.461777 |
| GOTERM_BP_FAT | GO:0002764~immune response-regulating signal transduction | 0.005866 | 1 | 10.53644 |
| GOTERM_BP_FAT | GO:0032259~methylation | 0.005977 | 1 | 10.72578 |
| GOTERM_BP_FAT | GO:0045892~negative regulation of transcription, DNA-dependent | 0.006113 | 1 | 10.95635 |
| GOTERM_BP_FAT | GO:0046356~acetyl-CoA catabolic process | 0.006158 | 1 | 11.03245 |
| GOTERM_BP_FAT | GO:0006099~tricarboxylic acid cycle | 0.006158 | 1 | 11.03245 |
| GOTERM_CC_FAT | GO:0005792~microsome | 0.006375 | 0.990737342 | 9.247161 |
| GOTERM_MF_FAT | GO:0004713~protein tyrosine kinase activity | 0.006628 | 0.999991117 | 10.64465 |
| GOTERM_CC_FAT | GO:0044437~vacuolar part | 0.006658 | 0.992479111 | 9.638114 |
| GOTERM_BP_FAT | GO:0031333~negative regulation of protein complex assembly | 0.007014 | 1 | 12.47277 |
| GOTERM_BP_FAT | GO:0051493~regulation of cytoskeleton organization | 0.007056 | 1 | 12.54164 |
| GOTERM_CC_FAT | GO:0005770~late endosome | 0.007104 | 0.994584344 | 10.25102 |
| GOTERM_BP_FAT | GO:0051169~nuclear transport | 0.007228 | 1 | 12.82921 |
| GOTERM_CC_FAT | GO:0031410~cytoplasmic vesicle | 0.007307 | 0.995337551 | 10.52914 |
| GOTERM_BP_FAT | GO:0022618~ribonucleoprotein complex assembly | 0.007309 | 1 | 12.96353 |
| GOTERM_MF_FAT | GO:0004529~exodeoxyribonuclease activity | 0.007348 | 0.9999975 | 11.73426 |
| GOTERM_MF_FAT | GO:0016895~exodeoxyribonuclease activity, producing 5'-phosphomonoesters | 0.007348 | 0.9999975 | 11.73426 |
| GOTERM_BP_FAT | GO:0009262~deoxyribonucleotide metabolic process | 0.00742 | 1 | 13.14657 |
| GOTERM_BP_FAT | GO:0050867~positive regulation of cell activation | 0.007833 | 1 | 13.82782 |
| GOTERM_BP_FAT | GO:0031647~regulation of protein stability | 0.008105 | 1 | 14.27458 |
| GOTERM_BP_FAT | GO:0002768~immune response-regulating cell surface receptor signaling pathway | 0.008105 | 1 | 14.27458 |
| GOTERM_BP_FAT | GO:0006266~DNA ligation | 0.008312 | 1 | 14.61266 |
| GOTERM_CC_FAT | GO:0000502~proteasome complex | 0.00837 | 0.997871707 | 11.97157 |
| GOTERM_BP_FAT | GO:0051187~cofactor catabolic process | 0.008446 | 1 | 14.831 |
| GOTERM_CC_FAT | GO:0016592~Srb-mediator complex | 0.008665 | 0.998288471 | 12.36827 |
| GOTERM_CC_FAT | GO:0005720~nuclear heterochromatin | 0.008665 | 0.998288471 | 12.36827 |
| GOTERM_BP_FAT | GO:0044087~regulation of cellular component biogenesis | 0.00868 | 1 | 15.20963 |
| GOTERM_BP_FAT | GO:0051129~negative regulation of cellular component organization | 0.00868 | 1 | 15.20963 |
| GOTERM_BP_FAT | GO:0019884~antigen processing and presentation of exogenous antigen | 0.00873 | 1 | 15.29098 |
| GOTERM_BP_FAT | GO:0006261~DNA-dependent DNA replication | 0.008915 | 1 | 15.58877 |
| GOTERM_BP_FAT | GO:0002478~antigen processing and presentation of exogenous peptide antigen | 0.00903 | 1 | 15.77396 |
| GOTERM_MF_FAT | GO:0008017~microtubule binding | 0.009049 | 0.999999875 | 14.25935 |
| GOTERM_BP_FAT | GO:0051253~negative regulation of RNA metabolic process | 0.009638 | 1 | 16.74706 |
| GOTERM_CC_FAT | GO:0000803~sex chromosome | 0.00984 | 0.999281599 | 13.93083 |
| GOTERM_MF_FAT | GO:0042623~ATPase activity, coupled | 0.009907 | 0.999999973 | 15.50663 |
| GOTERM_MF_FAT | GO:0042287~MHC protein binding | 0.01003 | 0.999999978 | 15.68485 |
| GOTERM_BP_FAT | GO:0001824~blastocyst development | 0.010303 | 1 | 17.79979 |
| GOTERM_BP_FAT | GO:0007030~Golgi organization | 0.010326 | 1 | 17.83519 |
| GOTERM_BP_FAT | GO:0009394~2'-deoxyribonucleotide metabolic process | 0.010326 | 1 | 17.83519 |
| GOTERM_BP_FAT | GO:0002709~regulation of T cell mediated immunity | 0.010326 | 1 | 17.83519 |
| GOTERM_BP_FAT | GO:0010608~posttranscriptional regulation of gene expression | 0.010891 | 1 | 18.71797 |
| GOTERM_BP_FAT | GO:0015980~energy derivation by oxidation of organic compounds | 0.01103 | 1 | 18.935 |
| GOTERM_MF_FAT | GO:0016455~RNA polymerase II transcription mediator activity | 0.01108 | 0.999999997 | 17.18537 |
| GOTERM_BP_FAT | GO:0045582~positive regulation of T cell differentiation | 0.01122 | 1 | 19.22843 |
| GOTERM_CC_FAT | GO:0000123~histone acetyltransferase complex | 0.011315 | 0.999758857 | 15.85623 |
| GOTERM_BP_FAT | GO:0032269~negative regulation of cellular protein metabolic process | 0.011549 | 1 | 19.73493 |
| GOTERM_CC_FAT | GO:0005795~Golgi stack | 0.011621 | 0.999807744 | 16.2504 |
| GOTERM_CC_FAT | GO:0048770~pigment granule | 0.011657 | 0.99981277 | 16.29637 |
| GOTERM_CC_FAT | GO:0042470~melanosome | 0.011657 | 0.99981277 | 16.29637 |
| GOTERM_CC_FAT | GO:0005667~transcription factor complex | 0.01166 | 0.999813151 | 16.29991 |
| GOTERM_BP_FAT | GO:0002366~leukocyte activation during immune response | 0.012012 | 1 | 20.44378 |
| GOTERM_BP_FAT | GO:0002263~cell activation during immune response | 0.012012 | 1 | 20.44378 |
| GOTERM_CC_FAT | GO:0044430~cytoskeletal part | 0.012052 | 0.99986024 | 16.8021 |
| GOTERM_BP_FAT | GO:0010033~response to organic substance | 0.012432 | 1 | 21.08132 |
| GOTERM_BP_FAT | GO:0033043~regulation of organelle organization | 0.012486 | 1 | 21.16359 |
| GOTERM_CC_FAT | GO:0016023~cytoplasmic membrane-bounded vesicle | 0.012728 | 0.999915352 | 17.66223 |
| GOTERM_MF_FAT | GO:0015631~tubulin binding | 0.012812 | 1 | 19.60533 |
| GOTERM_BP_FAT | GO:0032103~positive regulation of response to external stimulus | 0.012953 | 1 | 21.86586 |
| GOTERM_BP_FAT | GO:0022904~respiratory electron transport chain | 0.012953 | 1 | 21.86586 |
| GOTERM_BP_FAT | GO:0019220~regulation of phosphate metabolic process | 0.012982 | 1 | 21.90987 |
| GOTERM_BP_FAT | GO:0051174~regulation of phosphorus metabolic process | 0.012982 | 1 | 21.90987 |
| GOTERM_BP_FAT | GO:0005996~monosaccharide metabolic process | 0.012994 | 1 | 21.92801 |
| GOTERM_MF_FAT | GO:0030145~manganese ion binding | 0.013155 | 1 | 20.07783 |
| GOTERM_BP_FAT | GO:0002757~immune response-activating signal transduction | 0.013244 | 1 | 22.30051 |
| GOTERM_CC_FAT | GO:0005740~mitochondrial envelope | 0.013287 | 0.999944076 | 18.36654 |
| GOTERM_MF_FAT | GO:0043566~structure-specific DNA binding | 0.013306 | 1 | 20.2838 |
| GOTERM_CC_FAT | GO:0019866~organelle inner membrane | 0.013348 | 0.999946531 | 18.44247 |
| GOTERM_BP_FAT | GO:0043161~proteasomal ubiquitin-dependent protein catabolic process | 0.013379 | 1 | 22.50214 |
| GOTERM_BP_FAT | GO:0010498~proteasomal protein catabolic process | 0.013379 | 1 | 22.50214 |
| GOTERM_CC_FAT | GO:0031966~mitochondrial membrane | 0.013477 | 0.999951411 | 18.60408 |
| GOTERM_BP_FAT | GO:0032272~negative regulation of protein polymerization | 0.013607 | 1 | 22.83991 |
| GOTERM_BP_FAT | GO:0050773~regulation of dendrite development | 0.013716 | 1 | 23.00124 |
| GOTERM_BP_FAT | GO:0051094~positive regulation of developmental process | 0.013781 | 1 | 23.09658 |
| GOTERM_BP_FAT | GO:0051338~regulation of transferase activity | 0.01403 | 1 | 23.46359 |
| GOTERM_CC_FAT | GO:0031982~vesicle | 0.014101 | 0.999969434 | 19.38225 |
| GOTERM_MF_FAT | GO:0016887~ATPase activity | 0.014204 | 1 | 21.50283 |
| GOTERM_BP_FAT | GO:0006903~vesicle targeting | 0.014578 | 1 | 24.26464 |
| GOTERM_BP_FAT | GO:0051693~actin filament capping | 0.014578 | 1 | 24.26464 |
| GOTERM_BP_FAT | GO:0050851~antigen receptor-mediated signaling pathway | 0.014644 | 1 | 24.36133 |
| GOTERM_BP_FAT | GO:0002504~antigen processing and presentation of peptide or polysaccharide antigen via MHC class II | 0.014644 | 1 | 24.36133 |
| GOTERM_BP_FAT | GO:0045639~positive regulation of myeloid cell differentiation | 0.014644 | 1 | 24.36133 |
| GOTERM_BP_FAT | GO:0051248~negative regulation of protein metabolic process | 0.01483 | 1 | 24.63139 |
| GOTERM_BP_FAT | GO:0006511~ubiquitin-dependent protein catabolic process | 0.015 | 1 | 24.87584 |
| GOTERM_BP_FAT | GO:0070302~regulation of stress-activated protein kinase signaling pathway | 0.015102 | 1 | 25.02358 |
| GOTERM_CC_FAT | GO:0005743~mitochondrial inner membrane | 0.015169 | 0.999986171 | 20.69659 |
| GOTERM_MF_FAT | GO:0008047~enzyme activator activity | 0.015246 | 1 | 22.89495 |
| GOTERM_BP_FAT | GO:0046328~regulation of JNK cascade | 0.015448 | 1 | 25.52058 |
| GOTERM_CC_FAT | GO:0044450~microtubule organizing center part | 0.015946 | 0.999992241 | 21.6408 |
| GOTERM_CC_FAT | GO:0005774~vacuolar membrane | 0.015946 | 0.999992241 | 21.6408 |
| GOTERM_BP_FAT | GO:0006944~membrane fusion | 0.016127 | 1 | 26.48698 |
| GOTERM_MF_FAT | GO:0060090~molecular adaptor activity | 0.016216 | 1 | 24.17029 |
| GOTERM_BP_FAT | GO:0009109~coenzyme catabolic process | 0.016498 | 1 | 27.00989 |
| GOTERM_BP_FAT | GO:0051101~regulation of DNA binding | 0.016504 | 1 | 27.01847 |
| GOTERM_MF_FAT | GO:0019901~protein kinase binding | 0.016565 | 1 | 24.62516 |
| GOTERM_BP_FAT | GO:0051099~positive regulation of binding | 0.016734 | 1 | 27.34058 |
| GOTERM_BP_FAT | GO:0031397~negative regulation of protein ubiquitination | 0.017281 | 1 | 28.10207 |
| GOTERM_BP_FAT | GO:0051249~regulation of lymphocyte activation | 0.017304 | 1 | 28.13363 |
| GOTERM_BP_FAT | GO:0008154~actin polymerization or depolymerization | 0.017343 | 1 | 28.18656 |
| GOTERM_BP_FAT | GO:0031399~regulation of protein modification process | 0.017391 | 1 | 28.25322 |
| GOTERM_CC_FAT | GO:0000775~chromosome, centromeric region | 0.017407 | 0.999997384 | 23.38657 |
| GOTERM_BP_FAT | GO:0042325~regulation of phosphorylation | 0.017495 | 1 | 28.39711 |
| GOTERM_CC_FAT | GO:0044448~cell cortex part | 0.017787 | 0.999998029 | 23.8348 |
| GOTERM_BP_FAT | GO:0032663~regulation of interleukin-2 production | 0.017873 | 1 | 28.91688 |
| GOTERM_BP_FAT | GO:0045768~positive regulation of anti-apoptosis | 0.017873 | 1 | 28.91688 |
| GOTERM_BP_FAT | GO:0043549~regulation of kinase activity | 0.018322 | 1 | 29.52995 |
| GOTERM_BP_FAT | GO:0043085~positive regulation of catalytic activity | 0.018573 | 1 | 29.87013 |
| GOTERM_BP_FAT | GO:0016044~membrane organization | 0.018639 | 1 | 29.95813 |
| GOTERM_MF_FAT | GO:0030674~protein binding, bridging | 0.018756 | 1 | 27.41692 |
| GOTERM_BP_FAT | GO:0043648~dicarboxylic acid metabolic process | 0.018806 | 1 | 30.18395 |
| GOTERM_MF_FAT | GO:0042826~histone deacetylase binding | 0.018974 | 1 | 27.68924 |
| GOTERM_MF_FAT | GO:0051287~NAD or NADH binding | 0.019022 | 1 | 27.74868 |
| GOTERM_CC_FAT | GO:0031988~membrane-bounded vesicle | 0.019109 | 0.999999265 | 25.37555 |
| GOTERM_BP_FAT | GO:0034656~nucleobase, nucleoside and nucleotide catabolic process | 0.019156 | 1 | 30.65327 |
| GOTERM_BP_FAT | GO:0034655~nucleobase, nucleoside, nucleotide and nucleic acid catabolic process | 0.019156 | 1 | 30.65327 |
| GOTERM_MF_FAT | GO:0019887~protein kinase regulator activity | 0.019198 | 1 | 27.96865 |
| GOTERM_CC_FAT | GO:0015030~Cajal body | 0.019267 | 0.999999347 | 25.55815 |
| GOTERM_BP_FAT | GO:0045767~regulation of anti-apoptosis | 0.019387 | 1 | 30.96231 |
| GOTERM_BP_FAT | GO:0018108~peptidyl-tyrosine phosphorylation | 0.019776 | 1 | 31.47847 |
| GOTERM_BP_FAT | GO:0030111~regulation of Wnt receptor signaling pathway | 0.019776 | 1 | 31.47847 |
| GOTERM_CC_FAT | GO:0042105~alpha-beta T cell receptor complex | 0.019869 | 0.999999583 | 26.24776 |
| GOTERM_BP_FAT | GO:0009081~branched chain family amino acid metabolic process | 0.020448 | 1 | 32.36204 |
| GOTERM_MF_FAT | GO:0004386~helicase activity | 0.020864 | 1 | 30.01093 |
| GOTERM_BP_FAT | GO:0032268~regulation of cellular protein metabolic process | 0.020951 | 1 | 33.01659 |
| GOTERM_CC_FAT | GO:0044459~plasma membrane part | 0.021054 | 0.999999828 | 27.58927 |
| GOTERM_BP_FAT | GO:0044270~nitrogen compound catabolic process | 0.021557 | 1 | 33.79654 |
| GOTERM_BP_FAT | GO:0051444~negative regulation of ubiquitin-protein ligase activity | 0.021557 | 1 | 33.79654 |
| GOTERM_BP_FAT | GO:0051352~negative regulation of ligase activity | 0.021557 | 1 | 33.79654 |
| GOTERM_MF_FAT | GO:0000405~bubble DNA binding | 0.021798 | 1 | 31.13211 |
| GOTERM_MF_FAT | GO:0003696~satellite DNA binding | 0.021798 | 1 | 31.13211 |
| GOTERM_BP_FAT | GO:0006284~base-excision repair | 0.021839 | 1 | 34.15657 |
| GOTERM_BP_FAT | GO:0002200~somatic diversification of immune receptors | 0.021839 | 1 | 34.15657 |
| GOTERM_BP_FAT | GO:0042098~T cell proliferation | 0.021839 | 1 | 34.15657 |
| GOTERM_BP_FAT | GO:0001932~regulation of protein amino acid phosphorylation | 0.022156 | 1 | 34.55917 |
| GOTERM_BP_FAT | GO:0033365~protein localization in organelle | 0.022514 | 1 | 35.01154 |
| GOTERM_CC_FAT | GO:0005885~Arp2/3 protein complex | 0.022682 | 0.999999949 | 29.39534 |
| GOTERM_BP_FAT | GO:0051056~regulation of small GTPase mediated signal transduction | 0.023174 | 1 | 35.83723 |
| GOTERM_BP_FAT | GO:0050671~positive regulation of lymphocyte proliferation | 0.023328 | 1 | 36.02859 |
| GOTERM_BP_FAT | GO:0051336~regulation of hydrolase activity | 0.023495 | 1 | 36.23448 |
| GOTERM_BP_FAT | GO:0001779~natural killer cell differentiation | 0.023577 | 1 | 36.33677 |
| GOTERM_BP_FAT | GO:0045449~regulation of transcription | 0.023586 | 1 | 36.34679 |
| GOTERM_BP_FAT | GO:0019318~hexose metabolic process | 0.023644 | 1 | 36.41899 |
| GOTERM_BP_FAT | GO:0045086~positive regulation of interleukin-2 biosynthetic process | 0.023729 | 1 | 36.52372 |
| GOTERM_BP_FAT | GO:0046640~regulation of alpha-beta T cell proliferation | 0.023729 | 1 | 36.52372 |
| GOTERM_BP_FAT | GO:0045061~thymic T cell selection | 0.023729 | 1 | 36.52372 |
| GOTERM_BP_FAT | GO:0042100~B cell proliferation | 0.023729 | 1 | 36.52372 |
| GOTERM_BP_FAT | GO:0009166~nucleotide catabolic process | 0.023765 | 1 | 36.56745 |
| GOTERM_BP_FAT | GO:0045621~positive regulation of lymphocyte differentiation | 0.023791 | 1 | 36.59931 |
| GOTERM_BP_FAT | GO:0002429~immune response-activating cell surface receptor signaling pathway | 0.024149 | 1 | 37.039 |
| GOTERM_BP_FAT | GO:0042102~positive regulation of T cell proliferation | 0.024149 | 1 | 37.039 |
| GOTERM_CC_FAT | GO:0042613~MHC class II protein complex | 0.024589 | 0.999999988 | 31.45577 |
| GOTERM_MF_FAT | GO:0047485~protein N-terminus binding | 0.024657 | 1 | 34.46113 |
| GOTERM_MF_FAT | GO:0016879~ligase activity, forming carbon-nitrogen bonds | 0.024705 | 1 | 34.51539 |
| GOTERM_BP_FAT | GO:0009100~glycoprotein metabolic process | 0.025332 | 1 | 38.46785 |
| GOTERM_MF_FAT | GO:0016018~cyclosporin A binding | 0.025423 | 1 | 35.32645 |
| GOTERM_BP_FAT | GO:0051789~response to protein stimulus | 0.025475 | 1 | 38.6379 |
| GOTERM_MF_FAT | GO:0008094~DNA-dependent ATPase activity | 0.02562 | 1 | 35.54755 |
| GOTERM_BP_FAT | GO:0051340~regulation of ligase activity | 0.025985 | 1 | 39.24325 |
| GOTERM_BP_FAT | GO:0042127~regulation of cell proliferation | 0.026553 | 1 | 39.9104 |
| GOTERM_CC_FAT | GO:0001739~sex chromatin | 0.026581 | 0.999999997 | 33.54912 |
| GOTERM_CC_FAT | GO:0033276~transcription factor TFTC complex | 0.026581 | 0.999999997 | 33.54912 |
| GOTERM_BP_FAT | GO:0051603~proteolysis involved in cellular protein catabolic process | 0.026673 | 1 | 40.0505 |
| GOTERM_MF_FAT | GO:0051015~actin filament binding | 0.026693 | 1 | 36.73825 |
| GOTERM_BP_FAT | GO:0033151~V(D)J recombination | 0.026697 | 1 | 40.07856 |
| GOTERM_BP_FAT | GO:0018279~protein amino acid N-linked glycosylation via asparagine | 0.026697 | 1 | 40.07856 |
| GOTERM_BP_FAT | GO:0018196~peptidyl-asparagine modification | 0.026697 | 1 | 40.07856 |
| GOTERM_BP_FAT | GO:0016444~somatic cell DNA recombination | 0.026715 | 1 | 40.09862 |
| GOTERM_BP_FAT | GO:0002562~somatic diversification of immune receptors via germline recombination within a single locus | 0.026715 | 1 | 40.09862 |
| GOTERM_BP_FAT | GO:0030835~negative regulation of actin filament depolymerization | 0.026715 | 1 | 40.09862 |
| GOTERM_BP_FAT | GO:0051090~regulation of transcription factor activity | 0.026753 | 1 | 40.14371 |
| GOTERM_BP_FAT | GO:0042542~response to hydrogen peroxide | 0.027732 | 1 | 41.27275 |
| GOTERM_BP_FAT | GO:0032946~positive regulation of mononuclear cell proliferation | 0.027732 | 1 | 41.27275 |
| GOTERM_BP_FAT | GO:0070665~positive regulation of leukocyte proliferation | 0.027732 | 1 | 41.27275 |
| GOTERM_CC_FAT | GO:0000139~Golgi membrane | 0.0278 | 0.999999999 | 34.80127 |
| GOTERM_BP_FAT | GO:0009223~pyrimidine deoxyribonucleotide catabolic process | 0.027994 | 1 | 41.57089 |
| GOTERM_BP_FAT | GO:0046426~negative regulation of JAK-STAT cascade | 0.027994 | 1 | 41.57089 |
| GOTERM_MF_FAT | GO:0003677~DNA binding | 0.028003 | 1 | 38.16378 |
| GOTERM_BP_FAT | GO:0051351~positive regulation of ligase activity | 0.02823 | 1 | 41.83887 |
| GOTERM_BP_FAT | GO:0030837~negative regulation of actin filament polymerization | 0.028328 | 1 | 41.95051 |
| GOTERM_BP_FAT | GO:0030705~cytoskeleton-dependent intracellular transport | 0.028441 | 1 | 42.07731 |
| GOTERM_BP_FAT | GO:0048584~positive regulation of response to stimulus | 0.028544 | 1 | 42.19376 |
| GOTERM_BP_FAT | GO:0043623~cellular protein complex assembly | 0.028554 | 1 | 42.20541 |
| GOTERM_BP_FAT | GO:0006006~glucose metabolic process | 0.02887 | 1 | 42.55982 |
| GOTERM_BP_FAT | GO:0045936~negative regulation of phosphate metabolic process | 0.029039 | 1 | 42.74865 |
| GOTERM_BP_FAT | GO:0010563~negative regulation of phosphorus metabolic process | 0.029039 | 1 | 42.74865 |
| GOTERM_BP_FAT | GO:0018212~peptidyl-tyrosine modification | 0.029039 | 1 | 42.74865 |
| GOTERM_BP_FAT | GO:0030258~lipid modification | 0.02937 | 1 | 43.11703 |
| GOTERM_BP_FAT | GO:0051726~regulation of cell cycle | 0.029576 | 1 | 43.34473 |
| GOTERM_MF_FAT | GO:0019209~kinase activator activity | 0.029622 | 1 | 39.88387 |
| GOTERM_BP_FAT | GO:0046634~regulation of alpha-beta T cell activation | 0.02968 | 1 | 43.45985 |
| GOTERM_BP_FAT | GO:0030521~androgen receptor signaling pathway | 0.02968 | 1 | 43.45985 |
| GOTERM_CC_FAT | GO:0005881~cytoplasmic microtubule | 0.029706 | 1 | 36.71298 |
| GOTERM_MF_FAT | GO:0019210~kinase inhibitor activity | 0.030078 | 1 | 40.36015 |
| GOTERM_BP_FAT | GO:0051436~negative regulation of ubiquitin-protein ligase activity during mitotic cell cycle | 0.03051 | 1 | 44.36775 |
| GOTERM_BP_FAT | GO:0044257~cellular protein catabolic process | 0.031014 | 1 | 44.91349 |
| GOTERM_MF_FAT | GO:0008026~ATP-dependent helicase activity | 0.03113 | 1 | 41.4459 |
| GOTERM_MF_FAT | GO:0070035~purine NTP-dependent helicase activity | 0.03113 | 1 | 41.4459 |
| GOTERM_BP_FAT | GO:0051438~regulation of ubiquitin-protein ligase activity | 0.031171 | 1 | 45.08137 |
| GOTERM_MF_FAT | GO:0004518~nuclease activity | 0.031246 | 1 | 41.56467 |
| GOTERM_BP_FAT | GO:0008284~positive regulation of cell proliferation | 0.032238 | 1 | 46.2152 |
| GOTERM_BP_FAT | GO:0006333~chromatin assembly or disassembly | 0.032481 | 1 | 46.47042 |
| GOTERM_BP_FAT | GO:0000122~negative regulation of transcription from RNA polymerase II promoter | 0.032607 | 1 | 46.6024 |
| GOTERM_BP_FAT | GO:0030101~natural killer cell activation | 0.032715 | 1 | 46.71484 |
| GOTERM_BP_FAT | GO:0048284~organelle fusion | 0.032715 | 1 | 46.71484 |
| GOTERM_BP_FAT | GO:0002684~positive regulation of immune system process | 0.033235 | 1 | 47.25384 |
| GOTERM_BP_FAT | GO:0046578~regulation of Ras protein signal transduction | 0.033448 | 1 | 47.47359 |
| GOTERM_MF_FAT | GO:0003924~GTPase activity | 0.033577 | 1 | 43.89919 |
| GOTERM_BP_FAT | GO:0001889~liver development | 0.033765 | 1 | 47.79906 |
| GOTERM_BP_FAT | GO:0051443~positive regulation of ubiquitin-protein ligase activity | 0.033995 | 1 | 48.03305 |
| GOTERM_BP_FAT | GO:0010975~regulation of neuron projection development | 0.033995 | 1 | 48.03305 |
| GOTERM_BP_FAT | GO:0032844~regulation of homeostatic process | 0.034184 | 1 | 48.22591 |
| GOTERM_BP_FAT | GO:0045597~positive regulation of cell differentiation | 0.034217 | 1 | 48.25938 |
| GOTERM_BP_FAT | GO:0048285~organelle fission | 0.034217 | 1 | 48.25938 |
| GOTERM_BP_FAT | GO:0001910~regulation of leukocyte mediated cytotoxicity | 0.034886 | 1 | 48.93283 |
| GOTERM_BP_FAT | GO:0050772~positive regulation of axonogenesis | 0.034886 | 1 | 48.93283 |
| GOTERM_BP_FAT | GO:0000279~M phase | 0.035278 | 1 | 49.32396 |
| GOTERM_BP_FAT | GO:0042326~negative regulation of phosphorylation | 0.035537 | 1 | 49.581 |
| GOTERM_BP_FAT | GO:0043281~regulation of caspase activity | 0.035686 | 1 | 49.72819 |
| GOTERM_BP_FAT | GO:0043242~negative regulation of protein complex disassembly | 0.036174 | 1 | 50.20744 |
| GOTERM_BP_FAT | GO:0006406~mRNA export from nucleus | 0.036566 | 1 | 50.58921 |
| GOTERM_BP_FAT | GO:0051170~nuclear import | 0.036844 | 1 | 50.85903 |
| GOTERM_BP_FAT | GO:0006302~double-strand break repair | 0.036902 | 1 | 50.91467 |
| GOTERM_MF_FAT | GO:0008308~voltage-gated anion channel activity | 0.037162 | 1 | 47.31903 |
| GOTERM_BP_FAT | GO:0030163~protein catabolic process | 0.037645 | 1 | 51.62637 |
| GOTERM_CC_FAT | GO:0000313~organellar ribosome | 0.037876 | 1 | 44.33266 |
| GOTERM_CC_FAT | GO:0005761~mitochondrial ribosome | 0.037876 | 1 | 44.33266 |
| GOTERM_CC_FAT | GO:0070469~respiratory chain | 0.038131 | 1 | 44.55565 |
| GOTERM_BP_FAT | GO:0016197~endosome transport | 0.038356 | 1 | 52.29786 |
| GOTERM_CC_FAT | GO:0042645~mitochondrial nucleoid | 0.038799 | 1 | 45.13769 |
| GOTERM_CC_FAT | GO:0009295~nucleoid | 0.038799 | 1 | 45.13769 |
| GOTERM_MF_FAT | GO:0042802~identical protein binding | 0.038835 | 1 | 48.84671 |
| GOTERM_BP_FAT | GO:0045859~regulation of protein kinase activity | 0.038944 | 1 | 52.84773 |
| GOTERM_BP_FAT | GO:0051439~regulation of ubiquitin-protein ligase activity during mitotic cell cycle | 0.039138 | 1 | 53.02709 |
| GOTERM_BP_FAT | GO:0043632~modification-dependent macromolecule catabolic process | 0.039746 | 1 | 53.58633 |
| GOTERM_BP_FAT | GO:0019941~modification-dependent protein catabolic process | 0.039746 | 1 | 53.58633 |
| GOTERM_BP_FAT | GO:0002763~positive regulation of myeloid leukocyte differentiation | 0.040097 | 1 | 53.90663 |
| GOTERM_BP_FAT | GO:0002285~lymphocyte activation during immune response | 0.040097 | 1 | 53.90663 |
| GOTERM_BP_FAT | GO:0006516~glycoprotein catabolic process | 0.040097 | 1 | 53.90663 |
| GOTERM_BP_FAT | GO:0042770~DNA damage response, signal transduction | 0.040669 | 1 | 54.4238 |
| GOTERM_BP_FAT | GO:0030029~actin filament-based process | 0.041373 | 1 | 55.05225 |
| GOTERM_BP_FAT | GO:0001558~regulation of cell growth | 0.041377 | 1 | 55.05628 |
| GOTERM_BP_FAT | GO:0006595~polyamine metabolic process | 0.041421 | 1 | 55.09476 |
| GOTERM_MF_FAT | GO:0003779~actin binding | 0.041525 | 1 | 51.21605 |
| GOTERM_BP_FAT | GO:0032956~regulation of actin cytoskeleton organization | 0.041649 | 1 | 55.29701 |
| GOTERM_BP_FAT | GO:0030335~positive regulation of cell migration | 0.041649 | 1 | 55.29701 |
| GOTERM_BP_FAT | GO:0051272~positive regulation of cell motion | 0.042197 | 1 | 55.77818 |
| GOTERM_BP_FAT | GO:0045454~cell redox homeostasis | 0.042782 | 1 | 56.28648 |
| GOTERM_CC_FAT | GO:0000805~X chromosome | 0.043076 | 1 | 48.72616 |
| GOTERM_MF_FAT | GO:0016805~dipeptidase activity | 0.043498 | 1 | 52.88787 |
| GOTERM_BP_FAT | GO:0051591~response to cAMP | 0.043563 | 1 | 56.95637 |
| GOTERM_BP_FAT | GO:0006120~mitochondrial electron transport, NADH to ubiquinone | 0.043563 | 1 | 56.95637 |
| GOTERM_MF_FAT | GO:0004697~protein kinase C activity | 0.043574 | 1 | 52.95171 |
| GOTERM_BP_FAT | GO:0031175~neuron projection development | 0.043699 | 1 | 57.07179 |
| GOTERM_BP_FAT | GO:0050769~positive regulation of neurogenesis | 0.044652 | 1 | 57.87483 |
| GOTERM_BP_FAT | GO:0002377~immunoglobulin production | 0.045178 | 1 | 58.31152 |
| GOTERM_BP_FAT | GO:0009101~glycoprotein biosynthetic process | 0.045714 | 1 | 58.7521 |
| GOTERM_BP_FAT | GO:0007059~chromosome segregation | 0.046145 | 1 | 59.10298 |
| GOTERM_BP_FAT | GO:0016569~covalent chromatin modification | 0.046453 | 1 | 59.35273 |
| GOTERM_MF_FAT | GO:0017056~structural constituent of nuclear pore | 0.047021 | 1 | 55.74013 |
| GOTERM_MF_FAT | GO:0005522~profilin binding | 0.047021 | 1 | 55.74013 |
| GOTERM_BP_FAT | GO:0051437~positive regulation of ubiquitin-protein ligase activity during mitotic cell cycle | 0.04703 | 1 | 59.81533 |
| GOTERM_BP_FAT | GO:0032271~regulation of protein polymerization | 0.04703 | 1 | 59.81533 |
| GOTERM_BP_FAT | GO:0000087~M phase of mitotic cell cycle | 0.047425 | 1 | 60.12985 |
| GOTERM_BP_FAT | GO:0045060~negative thymic T cell selection | 0.047675 | 1 | 60.32705 |
| GOTERM_BP_FAT | GO:0000154~rRNA modification | 0.047675 | 1 | 60.32705 |
| GOTERM_BP_FAT | GO:0006244~pyrimidine nucleotide catabolic process | 0.047675 | 1 | 60.32705 |
| GOTERM_BP_FAT | GO:0006465~signal peptide processing | 0.047675 | 1 | 60.32705 |
| GOTERM_BP_FAT | GO:0002495~antigen processing and presentation of peptide antigen via MHC class II | 0.047675 | 1 | 60.32705 |
| GOTERM_BP_FAT | GO:0019886~antigen processing and presentation of exogenous peptide antigen via MHC class II | 0.047675 | 1 | 60.32705 |
| GOTERM_BP_FAT | GO:0048147~negative regulation of fibroblast proliferation | 0.047675 | 1 | 60.32705 |
| GOTERM_BP_FAT | GO:0045576~mast cell activation | 0.047675 | 1 | 60.32705 |
| GOTERM_BP_FAT | GO:0006285~base-excision repair, AP site formation | 0.047675 | 1 | 60.32705 |
| GOTERM_CC_FAT | GO:0031513~nonmotile primary cilium | 0.047678 | 1 | 52.34216 |
| GOTERM_CC_FAT | GO:0042101~T cell receptor complex | 0.047902 | 1 | 52.51202 |
| GOTERM_MF_FAT | GO:0016891~endoribonuclease activity, producing 5'-phosphomonoesters | 0.048037 | 1 | 56.53251 |
| GOTERM_MF_FAT | GO:0003727~single-stranded RNA binding | 0.048037 | 1 | 56.53251 |
| GOTERM_BP_FAT | GO:0043244~regulation of protein complex disassembly | 0.0484 | 1 | 60.89497 |
| GOTERM_CC_FAT | GO:0000776~kinetochore | 0.049037 | 1 | 53.36389 |
| GOTERM_BP_FAT | GO:0002711~positive regulation of T cell mediated immunity | 0.049153 | 1 | 61.4765 |
| GOTERM_BP_FAT | GO:0052547~regulation of peptidase activity | 0.049447 | 1 | 61.70129 |
| GOTERM_BP_FAT | GO:0010604~positive regulation of macromolecule metabolic process | 0.049527 | 1 | 61.76177 |
| GOTERM_CC_FAT | GO:0042612~MHC class I protein complex | 0.049813 | 1 | 53.93766 |
| GOTERM_BP_FAT | GO:0000280~nuclear division | 0.049972 | 1 | 62.09946 |
| GOTERM_BP_FAT | GO:0007067~mitosis | 0.049972 | 1 | 62.09946 |
| GOTERM_BP_FAT | GO:0006096~glycolysis | 0.05022 | 1 | 62.28608 |
| GOTERM_BP_FAT | GO:0031346~positive regulation of cell projection organization | 0.05022 | 1 | 62.28608 |
| GOTERM_BP_FAT | GO:0000389~nuclear mRNA 3'-splice site recognition | 0.050636 | 1 | 62.59748 |
| GOTERM_BP_FAT | GO:0045008~depyrimidination | 0.050636 | 1 | 62.59748 |
| GOTERM_BP_FAT | GO:0006346~methylation-dependent chromatin silencing | 0.050636 | 1 | 62.59748 |
| GOTERM_BP_FAT | GO:0032020~ISG15-protein conjugation | 0.050636 | 1 | 62.59748 |
| GOTERM_BP_FAT | GO:0051320~S phase | 0.050636 | 1 | 62.59748 |
| GOTERM_BP_FAT | GO:0000084~S phase of mitotic cell cycle | 0.050636 | 1 | 62.59748 |
| GOTERM_BP_FAT | GO:0050435~beta-amyloid metabolic process | 0.050636 | 1 | 62.59748 |
| GOTERM_BP_FAT | GO:0006573~valine metabolic process | 0.050636 | 1 | 62.59748 |
| GOTERM_BP_FAT | GO:0045136~development of secondary sexual characteristics | 0.050636 | 1 | 62.59748 |
| GOTERM_BP_FAT | GO:0042026~protein refolding | 0.050636 | 1 | 62.59748 |
| GOTERM_CC_FAT | GO:0017053~transcriptional repressor complex | 0.051017 | 1 | 54.81539 |
| GOTERM_CC_FAT | GO:0005884~actin filament | 0.051017 | 1 | 54.81539 |
| GOTERM_MF_FAT | GO:0003729~mRNA binding | 0.051247 | 1 | 58.94704 |
| GOTERM_BP_FAT | GO:0043543~protein amino acid acylation | 0.051652 | 1 | 63.3483 |
| GOTERM_BP_FAT | GO:0034660~ncRNA metabolic process | 0.051857 | 1 | 63.49783 |
| GOTERM_BP_FAT | GO:0007162~negative regulation of cell adhesion | 0.051944 | 1 | 63.56092 |
| GOTERM_BP_FAT | GO:0052548~regulation of endopeptidase activity | 0.052134 | 1 | 63.69909 |
| GOTERM_BP_FAT | GO:0000075~cell cycle checkpoint | 0.052615 | 1 | 64.04591 |
| GOTERM_MF_FAT | GO:0004527~exonuclease activity | 0.053354 | 1 | 60.46354 |
| GOTERM_BP_FAT | GO:0000724~double-strand break repair via homologous recombination | 0.053371 | 1 | 64.58492 |
| GOTERM_BP_FAT | GO:0000725~recombinational repair | 0.053371 | 1 | 64.58492 |
| GOTERM_BP_FAT | GO:0045058~T cell selection | 0.053371 | 1 | 64.58492 |
| GOTERM_BP_FAT | GO:0019320~hexose catabolic process | 0.053741 | 1 | 64.84594 |
| GOTERM_BP_FAT | GO:0042773~ATP synthesis coupled electron transport | 0.054053 | 1 | 65.06521 |
| GOTERM_BP_FAT | GO:0042775~mitochondrial ATP synthesis coupled electron transport | 0.054053 | 1 | 65.06521 |
| GOTERM_BP_FAT | GO:0032940~secretion by cell | 0.054434 | 1 | 65.33013 |
| GOTERM_MF_FAT | GO:0042277~peptide binding | 0.054542 | 1 | 61.29433 |
| GOTERM_BP_FAT | GO:0009060~aerobic respiration | 0.054808 | 1 | 65.58866 |
| GOTERM_BP_FAT | GO:0007265~Ras protein signal transduction | 0.055382 | 1 | 65.98201 |
| GOTERM_BP_FAT | GO:0030048~actin filament-based movement | 0.055393 | 1 | 65.98997 |
| GOTERM_MF_FAT | GO:0004860~protein kinase inhibitor activity | 0.055596 | 1 | 62.01819 |
| GOTERM_MF_FAT | GO:0030374~ligand-dependent nuclear receptor transcription coactivator activity | 0.055596 | 1 | 62.01819 |
| GOTERM_BP_FAT | GO:0051348~negative regulation of transferase activity | 0.055649 | 1 | 66.16389 |
| GOTERM_BP_FAT | GO:0022406~membrane docking | 0.055712 | 1 | 66.20619 |
| GOTERM_BP_FAT | GO:0008637~apoptotic mitochondrial changes | 0.055712 | 1 | 66.20619 |
| GOTERM_BP_FAT | GO:0002440~production of molecular mediator of immune response | 0.055712 | 1 | 66.20619 |
| GOTERM_BP_FAT | GO:0009306~protein secretion | 0.055712 | 1 | 66.20619 |
| GOTERM_CC_FAT | GO:0000790~nuclear chromatin | 0.055931 | 1 | 58.23725 |
| GOTERM_BP_FAT | GO:0030834~regulation of actin filament depolymerization | 0.056011 | 1 | 66.40838 |
| GOTERM_CC_FAT | GO:0030018~Z disc | 0.056028 | 1 | 58.3023 |
| GOTERM_BP_FAT | GO:0070085~glycosylation | 0.056205 | 1 | 66.53902 |
| GOTERM_BP_FAT | GO:0006486~protein amino acid glycosylation | 0.056205 | 1 | 66.53902 |
| GOTERM_BP_FAT | GO:0043413~biopolymer glycosylation | 0.056205 | 1 | 66.53902 |
| GOTERM_BP_FAT | GO:0006473~protein amino acid acetylation | 0.056492 | 1 | 66.73081 |
| GOTERM_BP_FAT | GO:0031570~DNA integrity checkpoint | 0.056492 | 1 | 66.73081 |
| GOTERM_BP_FAT | GO:0031145~anaphase-promoting complex-dependent proteasomal ubiquitin-dependent protein catabolic process | 0.056506 | 1 | 66.74035 |
| GOTERM_CC_FAT | GO:0016605~PML body | 0.057822 | 1 | 59.48895 |
| GOTERM_MF_FAT | GO:0000287~magnesium ion binding | 0.058169 | 1 | 63.73226 |
| GOTERM_BP_FAT | GO:0016055~Wnt receptor signaling pathway | 0.058251 | 1 | 67.88551 |
| GOTERM_BP_FAT | GO:0000226~microtubule cytoskeleton organization | 0.058763 | 1 | 68.2138 |
| GOTERM_BP_FAT | GO:0032970~regulation of actin filament-based process | 0.058812 | 1 | 68.24546 |
| GOTERM_BP_FAT | GO:0016573~histone acetylation | 0.058941 | 1 | 68.32787 |
| GOTERM_MF_FAT | GO:0005247~voltage-gated chloride channel activity | 0.059124 | 1 | 64.34935 |
| GOTERM_MF_FAT | GO:0048487~beta-tubulin binding | 0.059124 | 1 | 64.34935 |
| GOTERM_MF_FAT | GO:0008408~3'-5' exonuclease activity | 0.059576 | 1 | 64.63822 |
| GOTERM_BP_FAT | GO:0009219~pyrimidine deoxyribonucleotide metabolic process | 0.060162 | 1 | 69.09631 |
| GOTERM_BP_FAT | GO:0048814~regulation of dendrite morphogenesis | 0.060162 | 1 | 69.09631 |
| GOTERM_BP_FAT | GO:0006906~vesicle fusion | 0.060162 | 1 | 69.09631 |
| GOTERM_CC_FAT | GO:0045271~respiratory chain complex I | 0.06022 | 1 | 61.02512 |
| GOTERM_CC_FAT | GO:0030964~NADH dehydrogenase complex | 0.06022 | 1 | 61.02512 |
| GOTERM_CC_FAT | GO:0005747~mitochondrial respiratory chain complex I | 0.06022 | 1 | 61.02512 |
| GOTERM_BP_FAT | GO:0002694~regulation of leukocyte activation | 0.060382 | 1 | 69.23294 |
| GOTERM_CC_FAT | GO:0043190~ATP-binding cassette (ABC) transporter complex | 0.06047 | 1 | 61.18206 |
| GOTERM_CC_FAT | GO:0016600~flotillin complex | 0.06047 | 1 | 61.18206 |
| GOTERM_MF_FAT | GO:0016796~exonuclease activity, active with either ribo- or deoxyribonucleic acids and producing 5'-phosphomonoesters | 0.060846 | 1 | 65.43821 |
| GOTERM_BP_FAT | GO:0043388~positive regulation of DNA binding | 0.061097 | 1 | 69.67314 |
| GOTERM_CC_FAT | GO:0030131~clathrin adaptor complex | 0.061164 | 1 | 61.61476 |
| GOTERM_BP_FAT | GO:0007010~cytoskeleton organization | 0.061233 | 1 | 69.75642 |
| GOTERM_BP_FAT | GO:0006487~protein amino acid N-linked glycosylation | 0.061362 | 1 | 69.83488 |
| GOTERM_BP_FAT | GO:0048015~phosphoinositide-mediated signaling | 0.062169 | 1 | 70.32204 |
| GOTERM_CC_FAT | GO:0048471~perinuclear region of cytoplasm | 0.063854 | 1 | 63.24997 |
| GOTERM_MF_FAT | GO:0035257~nuclear hormone receptor binding | 0.065256 | 1 | 68.08452 |
| GOTERM_MF_FAT | GO:0051536~iron-sulfur cluster binding | 0.065615 | 1 | 68.29094 |
| GOTERM_MF_FAT | GO:0051540~metal cluster binding | 0.065615 | 1 | 68.29094 |
| GOTERM_MF_FAT | GO:0003684~damaged DNA binding | 0.065615 | 1 | 68.29094 |
| GOTERM_MF_FAT | GO:0032403~protein complex binding | 0.066173 | 1 | 68.61011 |
| GOTERM_CC_FAT | GO:0016581~NuRD complex | 0.066688 | 1 | 64.90209 |
| GOTERM_CC_FAT | GO:0035097~histone methyltransferase complex | 0.066688 | 1 | 64.90214 |
| GOTERM_CC_FAT | GO:0034708~methyltransferase complex | 0.066688 | 1 | 64.90214 |
| GOTERM_MF_FAT | GO:0034593~phosphatidylinositol bisphosphate phosphatase activity | 0.067072 | 1 | 69.11769 |
| GOTERM_MF_FAT | GO:0016864~intramolecular oxidoreductase activity, transposing S-S bonds | 0.067072 | 1 | 69.11769 |
| GOTERM_MF_FAT | GO:0004579~dolichyl-diphosphooligosaccharide-protein glycotransferase activity | 0.067072 | 1 | 69.11769 |
| GOTERM_MF_FAT | GO:0003756~protein disulfide isomerase activity | 0.067072 | 1 | 69.11769 |
| GOTERM_CC_FAT | GO:0034399~nuclear periphery | 0.067577 | 1 | 65.40559 |
| GOTERM_BP_FAT | GO:0001933~negative regulation of protein amino acid phosphorylation | 0.067736 | 1 | 73.48426 |
| GOTERM_BP_FAT | GO:0032886~regulation of microtubule-based process | 0.068632 | 1 | 73.96235 |
| GOTERM_BP_FAT | GO:0040017~positive regulation of locomotion | 0.068679 | 1 | 73.9876 |
| GOTERM_CC_FAT | GO:0042995~cell projection | 0.069057 | 1 | 66.22967 |
| GOTERM_BP_FAT | GO:0046365~monosaccharide catabolic process | 0.069121 | 1 | 74.21979 |
| GOTERM_BP_FAT | GO:0006986~response to unfolded protein | 0.069121 | 1 | 74.21979 |
| GOTERM_BP_FAT | GO:0031341~regulation of cell killing | 0.069124 | 1 | 74.22156 |
| GOTERM_CC_FAT | GO:0005788~endoplasmic reticulum lumen | 0.069307 | 1 | 66.36673 |
| GOTERM_BP_FAT | GO:0031344~regulation of cell projection organization | 0.06932 | 1 | 74.32434 |
| GOTERM_BP_FAT | GO:0006730~one-carbon metabolic process | 0.070413 | 1 | 74.88889 |
| GOTERM_BP_FAT | GO:0006007~glucose catabolic process | 0.071526 | 1 | 75.45184 |
| GOTERM_BP_FAT | GO:0007050~cell cycle arrest | 0.071693 | 1 | 75.53513 |
| GOTERM_BP_FAT | GO:0043433~negative regulation of transcription factor activity | 0.071854 | 1 | 75.61534 |
| GOTERM_BP_FAT | GO:0010551~regulation of specific transcription from RNA polymerase II promoter | 0.072715 | 1 | 76.04002 |
| GOTERM_BP_FAT | GO:0034504~protein localization in nucleus | 0.072715 | 1 | 76.04002 |
| GOTERM_BP_FAT | GO:0030174~regulation of DNA replication initiation | 0.073182 | 1 | 76.26715 |
| GOTERM_BP_FAT | GO:0000731~DNA synthesis during DNA repair | 0.073182 | 1 | 76.26715 |
| GOTERM_BP_FAT | GO:0050732~negative regulation of peptidyl-tyrosine phosphorylation | 0.073182 | 1 | 76.26715 |
| GOTERM_BP_FAT | GO:0042987~amyloid precursor protein catabolic process | 0.073182 | 1 | 76.26715 |
| GOTERM_BP_FAT | GO:0043383~negative T cell selection | 0.073182 | 1 | 76.26715 |
| GOTERM_BP_FAT | GO:0045047~protein targeting to ER | 0.073182 | 1 | 76.26715 |
| GOTERM_BP_FAT | GO:0033205~cytokinesis during cell cycle | 0.073182 | 1 | 76.26715 |
| GOTERM_MF_FAT | GO:0016779~nucleotidyltransferase activity | 0.073324 | 1 | 72.43916 |
| GOTERM_BP_FAT | GO:0046777~protein amino acid autophosphorylation | 0.07338 | 1 | 76.36299 |
| GOTERM_BP_FAT | GO:0017038~protein import | 0.073481 | 1 | 76.41166 |
| GOTERM_BP_FAT | GO:0044242~cellular lipid catabolic process | 0.073548 | 1 | 76.4442 |
| GOTERM_CC_FAT | GO:0030119~AP-type membrane coat adaptor complex | 0.074037 | 1 | 68.86848 |
| GOTERM_BP_FAT | GO:0006979~response to oxidative stress | 0.074517 | 1 | 76.90608 |
| GOTERM_CC_FAT | GO:0000125~PCAF complex | 0.074834 | 1 | 69.27289 |
| GOTERM_MF_FAT | GO:0051117~ATPase binding | 0.075086 | 1 | 73.31283 |
| GOTERM_BP_FAT | GO:0006405~RNA export from nucleus | 0.075087 | 1 | 77.17374 |
| GOTERM_BP_FAT | GO:0030036~actin cytoskeleton organization | 0.07513 | 1 | 77.19386 |
| GOTERM_BP_FAT | GO:0030833~regulation of actin filament polymerization | 0.07534 | 1 | 77.2917 |
| GOTERM_BP_FAT | GO:0002706~regulation of lymphocyte mediated immunity | 0.07534 | 1 | 77.2917 |
| GOTERM_BP_FAT | GO:0000079~regulation of cyclin-dependent protein kinase activity | 0.07534 | 1 | 77.2917 |
| GOTERM_MF_FAT | GO:0032947~protein complex scaffold | 0.075395 | 1 | 73.46315 |
| GOTERM_MF_FAT | GO:0051539~4 iron, 4 sulfur cluster binding | 0.075395 | 1 | 73.46315 |
| GOTERM_CC_FAT | GO:0030118~clathrin coat | 0.076068 | 1 | 69.88856 |
| GOTERM_CC_FAT | GO:0009986~cell surface | 0.076494 | 1 | 70.09877 |
| GOTERM_BP_FAT | GO:0033673~negative regulation of kinase activity | 0.077017 | 1 | 78.05851 |
| GOTERM_BP_FAT | GO:0030832~regulation of actin filament length | 0.077156 | 1 | 78.12104 |
| GOTERM_MF_FAT | GO:0005164~tumor necrosis factor receptor binding | 0.077229 | 1 | 74.3403 |
| GOTERM_MF_FAT | GO:0032393~MHC class I receptor activity | 0.077562 | 1 | 74.49639 |
| GOTERM_MF_FAT | GO:0000146~microfilament motor activity | 0.077562 | 1 | 74.49639 |
| GOTERM_BP_FAT | GO:0051259~protein oligomerization | 0.077578 | 1 | 78.30968 |
| GOTERM_BP_FAT | GO:0046164~alcohol catabolic process | 0.077705 | 1 | 78.36584 |
| GOTERM_BP_FAT | GO:0007015~actin filament organization | 0.077829 | 1 | 78.42099 |
| GOTERM_BP_FAT | GO:0043506~regulation of JUN kinase activity | 0.078266 | 1 | 78.61351 |
| GOTERM_MF_FAT | GO:0005504~fatty acid binding | 0.078454 | 1 | 74.91059 |
| GOTERM_MF_FAT | GO:0003714~transcription corepressor activity | 0.078503 | 1 | 74.93308 |
| GOTERM_CC_FAT | GO:0009897~external side of plasma membrane | 0.080377 | 1 | 71.95011 |
| GOTERM_BP_FAT | GO:0000165~MAPKKK cascade | 0.0804 | 1 | 79.5317 |
| GOTERM_BP_FAT | GO:0051260~protein homooligomerization | 0.080447 | 1 | 79.55132 |
| GOTERM_BP_FAT | GO:0006612~protein targeting to membrane | 0.081288 | 1 | 79.90245 |
| GOTERM_MF_FAT | GO:0004439~phosphatidylinositol-4,5-bisphosphate 5-phosphatase activity | 0.081288 | 1 | 76.18516 |
| GOTERM_MF_FAT | GO:0030332~cyclin binding | 0.081288 | 1 | 76.18516 |
| GOTERM_MF_FAT | GO:0070568~guanylyltransferase activity | 0.081288 | 1 | 76.18516 |
| GOTERM_MF_FAT | GO:0050733~RS domain binding | 0.081288 | 1 | 76.18516 |
| GOTERM_MF_FAT | GO:0016717~oxidoreductase activity, acting on paired donors, with oxidation of a pair of donors resulting in the reduction of molecular oxygen to two molecules of water | 0.081288 | 1 | 76.18516 |
| GOTERM_MF_FAT | GO:0034595~phosphoinositide 5-phosphatase activity | 0.081288 | 1 | 76.18516 |
| GOTERM_BP_FAT | GO:0051100~negative regulation of binding | 0.081526 | 1 | 80.00057 |
| GOTERM_BP_FAT | GO:0002237~response to molecule of bacterial origin | 0.081607 | 1 | 80.03391 |
| GOTERM_BP_FAT | GO:0006606~protein import into nucleus | 0.081607 | 1 | 80.03391 |
| GOTERM_BP_FAT | GO:0040029~regulation of gene expression, epigenetic | 0.082316 | 1 | 80.32375 |
| GOTERM_BP_FAT | GO:0046700~heterocycle catabolic process | 0.082316 | 1 | 80.32375 |
| GOTERM_BP_FAT | GO:0045730~respiratory burst | 0.082932 | 1 | 80.57238 |
| GOTERM_BP_FAT | GO:0002286~T cell activation during immune response | 0.082932 | 1 | 80.57238 |
| GOTERM_BP_FAT | GO:0051084~'de novo' posttranslational protein folding | 0.082932 | 1 | 80.57238 |
| GOTERM_MF_FAT | GO:0016893~endonuclease activity, active with either ribo- or deoxyribonucleic acids and producing 5'-phosphomonoesters | 0.083008 | 1 | 76.92852 |
| GOTERM_CC_FAT | GO:0005669~transcription factor TFIID complex | 0.083279 | 1 | 73.26346 |
| GOTERM_BP_FAT | GO:0050865~regulation of cell activation | 0.083386 | 1 | 80.75354 |
| GOTERM_BP_FAT | GO:0001816~cytokine production | 0.083444 | 1 | 80.77657 |
| GOTERM_BP_FAT | GO:0031396~regulation of protein ubiquitination | 0.083682 | 1 | 80.8708 |
| GOTERM_BP_FAT | GO:0048872~homeostasis of number of cells | 0.083682 | 1 | 80.8708 |
| GOTERM_BP_FAT | GO:0043393~regulation of protein binding | 0.083995 | 1 | 80.99415 |
| GOTERM_CC_FAT | GO:0005787~signal peptidase complex | 0.084701 | 1 | 73.88556 |
| GOTERM_MF_FAT | GO:0003743~translation initiation factor activity | 0.085744 | 1 | 78.06631 |
| GOTERM_MF_FAT | GO:0003704~specific RNA polymerase II transcription factor activity | 0.086104 | 1 | 78.21203 |
| GOTERM_BP_FAT | GO:0000186~activation of MAPKK activity | 0.086131 | 1 | 81.81567 |
| GOTERM_BP_FAT | GO:0043331~response to dsRNA | 0.086131 | 1 | 81.81567 |
| GOTERM_BP_FAT | GO:0007040~lysosome organization | 0.086131 | 1 | 81.81567 |
| GOTERM_BP_FAT | GO:0051494~negative regulation of cytoskeleton organization | 0.086143 | 1 | 81.81999 |
| GOTERM_BP_FAT | GO:0010639~negative regulation of organelle organization | 0.086508 | 1 | 81.95682 |
| GOTERM_CC_FAT | GO:0001772~immunological synapse | 0.086897 | 1 | 74.82004 |
| GOTERM_CC_FAT | GO:0008250~oligosaccharyltransferase complex | 0.086897 | 1 | 74.82004 |
| GOTERM_CC_FAT | GO:0031901~early endosome membrane | 0.086897 | 1 | 74.82004 |
| GOTERM_BP_FAT | GO:0006047~UDP-N-acetylglucosamine metabolic process | 0.087163 | 1 | 82.20007 |
| GOTERM_BP_FAT | GO:0019369~arachidonic acid metabolic process | 0.087163 | 1 | 82.20007 |
| GOTERM_BP_FAT | GO:0007252~I-kappaB phosphorylation | 0.087163 | 1 | 82.20007 |
| GOTERM_BP_FAT | GO:0048541~Peyer's patch development | 0.087163 | 1 | 82.20007 |
| GOTERM_BP_FAT | GO:0042532~negative regulation of tyrosine phosphorylation of STAT protein | 0.087163 | 1 | 82.20007 |
| GOTERM_BP_FAT | GO:0048537~mucosal-associated lymphoid tissue development | 0.087163 | 1 | 82.20007 |
| GOTERM_BP_FAT | GO:0006390~transcription from mitochondrial promoter | 0.087163 | 1 | 82.20007 |
| GOTERM_BP_FAT | GO:0000086~G2/M transition of mitotic cell cycle | 0.087271 | 1 | 82.24 |
| GOTERM_BP_FAT | GO:0051017~actin filament bundle formation | 0.087271 | 1 | 82.24 |
| GOTERM_BP_FAT | GO:0070507~regulation of microtubule cytoskeleton organization | 0.087697 | 1 | 82.39618 |
| GOTERM_BP_FAT | GO:0006888~ER to Golgi vesicle-mediated transport | 0.087697 | 1 | 82.39618 |
| GOTERM_BP_FAT | GO:0006412~translation | 0.089644 | 1 | 83.09373 |
| GOTERM_MF_FAT | GO:0043140~ATP-dependent 3'-5' DNA helicase activity | 0.09 | 1 | 79.73155 |
| GOTERM_MF_FAT | GO:0004525~ribonuclease III activity | 0.09 | 1 | 79.73155 |
| GOTERM_CC_FAT | GO:0005875~microtubule associated complex | 0.090433 | 1 | 76.2598 |
| GOTERM_BP_FAT | GO:0043392~negative regulation of DNA binding | 0.091016 | 1 | 83.56948 |
| GOTERM_CC_FAT | GO:0031965~nuclear membrane | 0.091147 | 1 | 76.54093 |
| GOTERM_MF_FAT | GO:0004437~inositol or phosphatidylinositol phosphatase activity | 0.09198 | 1 | 80.46499 |
| GOTERM_CC_FAT | GO:0070161~anchoring junction | 0.092288 | 1 | 76.98367 |
| GOTERM_BP_FAT | GO:0006839~mitochondrial transport | 0.092492 | 1 | 84.06724 |
| GOTERM_BP_FAT | GO:0002252~immune effector process | 0.094141 | 1 | 84.60639 |
| GOTERM_BP_FAT | GO:0001543~ovarian follicle rupture | 0.094744 | 1 | 84.79908 |
| GOTERM_BP_FAT | GO:0042524~negative regulation of tyrosine phosphorylation of Stat5 protein | 0.094744 | 1 | 84.79908 |
| GOTERM_BP_FAT | GO:0050862~positive regulation of T cell receptor signaling pathway | 0.094744 | 1 | 84.79908 |
| GOTERM_CC_FAT | GO:0005765~lysosomal membrane | 0.095147 | 1 | 78.05924 |
| GOTERM_MF_FAT | GO:0016862~intramolecular oxidoreductase activity, interconverting keto- and enol-groups | 0.095898 | 1 | 81.84383 |
| GOTERM_MF_FAT | GO:0004576~oligosaccharyl transferase activity | 0.095898 | 1 | 81.84383 |
| GOTERM_CC_FAT | GO:0005902~microvillus | 0.096215 | 1 | 78.44879 |
| GOTERM_BP_FAT | GO:0002708~positive regulation of lymphocyte mediated immunity | 0.096383 | 1 | 85.31174 |
| GOTERM_BP_FAT | GO:0002705~positive regulation of leukocyte mediated immunity | 0.096383 | 1 | 85.31174 |
| GOTERM_CC_FAT | GO:0005746~mitochondrial respiratory chain | 0.096799 | 1 | 78.65917 |
| GOTERM_BP_FAT | GO:0006338~chromatin remodeling | 0.097883 | 1 | 85.76627 |
| GOTERM_BP_FAT | GO:0034470~ncRNA processing | 0.098603 | 1 | 85.97965 |
| GOTERM_MF_FAT | GO:0008170~N-methyltransferase activity | 0.098627 | 1 | 82.74937 |
| GOTERM_MF_FAT | GO:0030295~protein kinase activator activity | 0.098856 | 1 | 82.82314 |
| GOTERM_MF_FAT | GO:0043022~ribosome binding | 0.098856 | 1 | 82.82314 |
| GOTERM_MF_FAT | GO:0016409~palmitoyltransferase activity | 0.099657 | 1 | 83.07974 |
| KEGG_PATHWAY | hsa05416:Viral myocarditis | 9.15E-06 | 0.001763879 | 0.011398 |
| KEGG_PATHWAY | hsa05330:Allograft rejection | 9.68E-06 | 0.001866543 | 0.012062 |
| KEGG_PATHWAY | hsa04940:Type I diabetes mellitus | 1.24E-05 | 0.002394783 | 0.015479 |
| KEGG_PATHWAY | hsa05332:Graft-versus-host disease | 4.63E-05 | 0.0088886 | 0.057628 |
| KEGG_PATHWAY | hsa04612:Antigen processing and presentation | 1.46E-04 | 0.027754308 | 0.181561 |
| KEGG_PATHWAY | hsa05320:Autoimmune thyroid disease | 1.64E-04 | 0.031137928 | 0.204026 |
| KEGG_PATHWAY | hsa03040:Spliceosome | 4.24E-04 | 0.078662864 | 0.52757 |
| KEGG_PATHWAY | hsa00020:Citrate cycle (TCA cycle) | 9.18E-04 | 0.162456999 | 1.138079 |
| KEGG_PATHWAY | hsa04660:T cell receptor signaling pathway | 0.001764 | 0.288775428 | 2.176096 |
| KEGG_PATHWAY | hsa04672:Intestinal immune network for IgA production | 0.001993 | 0.319595483 | 2.455494 |
| KEGG_PATHWAY | hsa05016:Huntington's disease | 0.008807 | 0.818648484 | 10.43728 |
| KEGG_PATHWAY | hsa04130:SNARE interactions in vesicular transport | 0.0101 | 0.859034278 | 11.88223 |
| KEGG_PATHWAY | hsa05340:Primary immunodeficiency | 0.011206 | 0.886377783 | 13.10053 |
| KEGG_PATHWAY | hsa04722:Neurotrophin signaling pathway | 0.012619 | 0.913782947 | 14.6354 |
| KEGG_PATHWAY | hsa05310:Asthma | 0.013633 | 0.929292105 | 15.72141 |
| KEGG_PATHWAY | hsa04666:Fc gamma R-mediated phagocytosis | 0.016327 | 0.958296463 | 18.54587 |
| KEGG_PATHWAY | hsa05010:Alzheimer's disease | 0.019343 | 0.976943661 | 21.60371 |
| KEGG_PATHWAY | hsa04144:Endocytosis | 0.021196 | 0.983993098 | 23.42919 |
| KEGG_PATHWAY | hsa05110:Vibrio cholerae infection | 0.024105 | 0.99098873 | 26.21753 |
| KEGG_PATHWAY | hsa04662:B cell receptor signaling pathway | 0.02537 | 0.992984593 | 27.40061 |
| KEGG_PATHWAY | hsa05220:Chronic myeloid leukemia | 0.02537 | 0.992984593 | 27.40061 |
| KEGG_PATHWAY | hsa04210:Apoptosis | 0.027461 | 0.995364615 | 29.31723 |
| KEGG_PATHWAY | hsa03022:Basal transcription factors | 0.027544 | 0.995440775 | 29.39279 |
| KEGG_PATHWAY | hsa04650:Natural killer cell mediated cytotoxicity | 0.037837 | 0.999415165 | 38.16031 |
| KEGG_PATHWAY | hsa04142:Lysosome | 0.037982 | 0.999431986 | 38.27672 |
| KEGG_PATHWAY | hsa04622:RIG-I-like receptor signaling pathway | 0.044414 | 0.999844386 | 43.22671 |
| KEGG_PATHWAY | hsa03018:RNA degradation | 0.054885 | 0.999981442 | 50.50994 |
| KEGG_PATHWAY | hsa04010:MAPK signaling pathway | 0.059968 | 0.999993446 | 53.72614 |
| KEGG_PATHWAY | hsa03440:Homologous recombination | 0.064829 | 0.99999759 | 56.62137 |
| KEGG_PATHWAY | hsa04070:Phosphatidylinositol signaling system | 0.067229 | 0.999998533 | 57.98833 |
| KEGG_PATHWAY | hsa04640:Hematopoietic cell lineage | 0.067881 | 0.999998718 | 58.35242 |
| KEGG_PATHWAY | hsa04920:Adipocytokine signaling pathway | 0.075647 | 0.999999745 | 62.476 |
| KEGG_PATHWAY | hsa05012:Parkinson's disease | 0.078426 | 0.999999857 | 63.85746 |
| KEGG_PATHWAY | hsa04062:Chemokine signaling pathway | 0.082849 | 0.999999944 | 65.96078 |
